# Metabolomics of early myocardial ischemia

**DOI:** 10.1007/s11306-023-01999-8

**Published:** 2023-04-01

**Authors:** Rasmus Bo Hasselbalch, Jonas Henrik Kristensen, Nina Strandkjær, Nicoline Jørgensen, Henning Bundgaard, Anders Malmendal, Kasper Karmark Iversen

**Affiliations:** 1 Department of Emergency Medicine, Copenhagen University Hospital, Herlev and Gentofte Hospital, Copenhagen, Denmark; 2 Department of Cardiology Medicine, Copenhagen University Hospital, Herlev and Gentofte Hospital, Copenhagen, Denmark; 3 Department of Cardiology, Copenhagen University Hospital, Rigshospitalet, Copenhagen, Denmark; 4 Department of Clinical Medicine, University of Copenhagen, Copenhagen, Denmark; 5 Department of Science and Environment, Roskilde University, Roskilde, Denmark; 6 Department of Emergency Medicine, Department of Cardiology, Herlev and Gentofte Hospital, Borgmester Ib Juuls vej 1, Herlev, DK-2730 Denmark

**Keywords:** Myocardial ischemia, Metabolomics, NMR, Coronary angiography, Myocardial infarction

## Abstract

**Introduction:**

Diagnosing myocardial infarction is difficult during the initial phase. As, acute myocardial ischemia is associated with changes in metabolic pathways, metabolomics may provide ways of identifying early stages of ischemia. We investigated the changes in metabolites after induced ischemia in humans using nuclear magnetic resonance spectroscopy (NMR).

**Methods:**

We included patients undergoing elective coronary angiography showing normal coronary arteries. These were randomized into 4 groups and underwent coronary artery occlusion for 0, 30, 60 or 90 s. Blood was collected over the next 3 h and analyzed using NMR. We used 2-way ANOVA of time from baseline- and treatment group to find metabolites that changed significantly following the intervention and principal component analysis (PCA) to investigate changes between the 90 s ischemia- and control groups at 15 and 60 min after intervention.

**Results:**

We included 34 patients. The most pronounced changes were observed in the lipid metabolism where 38 of 112 lipoprotein parameters (34%) showed a significant difference between the patients exposed to ischemia and the control group. There was a decrease in total plasma triglycerides over the first hour followed by a normalization. The principal component analysis showed a effects of the treatment after just 15 min. These effects were dominated by changes in high-density lipoprotein. An increase in lactic acid levels was detected surprisingly late, 1–2 h after the ischemia.

**Conclusion:**

We investigated the earliest changes in metabolites of patients undergoing brief myocardial ischemia and found that ischemia led to changes throughout the lipid metabolism as early as 15 min post-intervention.

**Supplementary Information:**

The online version contains supplementary material available at 10.1007/s11306-023-01999-8.

## Introduction

Chest pain is the primary complaint in 10% of patient visits in the emergency department (Plesner et al., 2015; Wibring et al., 2016). This can be a symptom of a wide range of illnesses from the non-emergent to acutely life-threatening conditions. While the number of patients with chest pain is rising, the proportion of patients with myocardial infarction (MI) seems to be falling (Wibring et al., 2016). This has made early diagnosis of MI an important focus in research. Cardiac troponins (cTn) are the biochemical gold standard of MI (Thygesen et al., 2019). While the increasing sensitivity of cTn assays have led to the possibility of earlier rule-out of myocardial ischemia, biomarkers are still occasionally within normal range for patients presenting early following ischemia necessitating further testing 1–3 h after admission (Collet et al., 2021).

Metabolomic phenotyping, or metabolomics for short, is the measurement of dynamic responses of a living system and can give unique insight into biological systems (Nicholson et al., 1999). Metabolomics has the potential to provide new means of diagnosing, monitoring and treating patients with MI and recent advances have made clinical implementation increasingly feasible (Bodi et al., 2013; McGarrah et al., 2018). Nuclear magnetic resonance (NMR) spectroscopy is a rapid and versatile technique for molecular and biophysical studies that quantifies all the most abundant metabolites such as organic acids, amino acids as well as larger units like lipoproteins in a cheap and highly reproducible way (Bothwell & Griffin, 2011; Mallol et al., 2013).

There have already been promising results for metabolomics in the cardiovascular field. A large study of metabolites in the blood of healthy adults found that the concentration of phosphatidylcholines had a strong inverse association to risk of coronary heart disease over multiple years of follow-up (Cavus et al., 2019). Similarly, a recent study found that the concentration of a subset of sphingolipids and phospholipids could be used to risk stratify patients for in-stent restenosis following percutaneous coronary intervention (PCI) with high accuracy(Cui et al., 2017). For acute cardiac ischemia and MI there have been multiple studies, but with little consensus yet. In clinical cohorts, there have been investigations of patients with coronary artery disease undergoing exercise stress testing (Sabatine et al., 2005), patients undergoing septal alcohol ablation (Lewis et al., 2008) and patients with ST-elevation MI and unstable angina (Ali et al., 2016). Maybe most promisingly, a study developed and validated a score consisting of metabolites, which was associated with coronary disease among patients with chest pain and a normal cTn (Bodi et al., 2012).

In a recent study (Árnadóttir et al., 2021), we reported the results of cTn and copeptin measurements in a model of balloon-induced ischemia in humans without coronary artery disease. Here we showed that as little as 30 s of ischemia led to an increase in cTn, which raised questions about the mechanisms of release for cTn as a previous study on animals showed no necrosis after 10 min of ischemia (Defilippi & Mills, 2021; Weil et al., 2017). In the present study, we used the same model of ischemia to investigate the temporal changes in plasma metabolites in humans using NMR spectroscopy.

## Methods

This was a prospective, randomized study of patients undergoing elective coronary angiography on suspicion of coronary artery disease from October 2016 to September 2017. The methods have been published previously (Árnadóttir et al., 2021). In brief, patients were included before undergoing a coronary angiography if they had no history of heart disease, renal failure or plasma creatinine > 100 mmol/L. If the coronary angiography was without any stenoses or atheromatosis, the patient was randomized to either the control group or to one of three ischemia groups; in the three groups the patients underwent balloon occlusion of the left anterior descending artery between the first and second diagonal branch for 30, 60 or 90 s. All patients were treated with intravenous heparin (70 IE/kg) and, when necessary due to chest pain, either intravenous fentanyl or nitroglycerin.

Blood samples were drawn every 15 min for the first 3 h, followed by every 30 min for the next 3 h from a peripheral venous catheter in the cubital vein and collected in serum or plasma tubes. For this study EDTA plasma was used. Samples were centrifuged for 10 min at 3,000 g aliquoted and stored at − 80 °C until analysis. There were no additional freeze-thaw cycles prior to analysis.

### Compliance with ethical standards

All procedures performed in studies involving human participants were in accordance with the ethical standards of the institutional and/or national research committee and with the 1964 Helsinki declaration and its later amendments or comparable ethical standards. The study was approved by the local Research Ethics Committee in Denmark (H-16,027,749) in accordance with Danish law and was registered at clinicaltrials.gov (identification number: NCT03203057). All included patients provided written informed consent.

### Sample preparation and 1 H NMR

All the samples were prepared using standard Bruker methods (Dona et al., 2014). Standard ^1^ H NMR spectra: NOESYGPPR1D and CPMG were recorded on a 600 MHz Avance III Bruker NMR spectrometer (Bruker Biospin, Rheinstetten, Germany) operating at 600.13 MHz as described previously (Kaluarachchi et al., 2018). All experiments were analyzed using the Bruker in vitro Diagnostics research methods, measuring 39 metabolite concentrations and 112 lipoprotein parameters (Jiménez et al., 2018).

### Statistics

Since this was an exploratory analysis with a limited number of participants, we chose to focus primarily on the differences between the 90 s ischemia- and control group. Data for the comparisons between 30 and 60 s ischemia groups and controls are available in supplementary data. Baseline levels of metabolites were presented as medians (interquartile range (IQR)). We used 2-way ANOVA of time from baseline- and treatment group with the raw concentrations and selected groups with both a significant difference in time and group to find metabolites that changed based on the intervention. These analyses were adjusted using the Hochberg-Benjamini method for controlling false discovery rates (Benjamini & Hochberg, 1995). The changes in metabolites were visualized using changes relative to baseline in percentages using smoothed conditional means with confidence intervals calculated using local polynomial regression fitting. Principal component analysis (PCA) was performed including all metabolites with no missing values (see supplementary Tables 1–2) comparing change in concentrations between the 90 s ischemia- and control groups at 15 and 60 min after the intervention. The 6 metabolites with the highest loadings in PC1 and 2 were investigated further visualized using boxplots of the concentration changes. Finally, since most of the 6 metabolites seemed to exhibit a threshold effect at 90 s ischemia, we tested the significance of the relative change in the 90 s group with all other participants. A p-value of < 0.05 was considered significant. Analyses were performed using R (version 4.1.0)(R Core Team, 2021) using the *tidyverse* package (Wickham et al., 2019).

## Results

A total of 34 patients were included in the study. Baseline characteristics of the cohort are presented in Table 1 with concentration of the lipid subclasses and selected metabolites at time 0. No significant differences between groups were present at baseline. Supplementary Table 3 shows the baseline concentrations of all metabolites.

**Table 1:** Baseline characteristics of the cohort. IQR – interquartile range, hs-cTnI – high sensitivity cardiac troponin I, LDL – Low Density Lipoprotein, HDL – High Density Lipoprotein, TCA cycle - Tricarboxylic Acid Cycle

	0 s ischemia	30 s ischemia	60 s ischemia	90 s ischemia
n	9	8	9	8
Age years, median (IQR)	62 (53–66)	61 (45–66)	55 (47–68)	60 (56–68)
Female sex, n (%)	5 (56)	5 (63)	4 (44)	5 (63)
**The main lipoprotein components, median (IQR)**				
Total Plasma Triglycerides (mg/dL)	66.12 (53.00-72.38)	80.35 (58.12–102.60)	81.95 (70.16- 147.64)	65.74 (54.94–77.01)
Total Plasma Cholesterol (mg/dL)	196.99 (176.46-212.02)	185.44 (168.69-232.39)	182.38 (173.89-219.73)	187.94 (180.83-213.93)
LDL Cholesterol (mg/dL)	108.18 (93.88-124.53)	103.08 (94.27-128.82)	83.24 (80.30-105.13)	102.47 (91.87-117.05)
HDL Cholesterol (mg/dL)	57.44 (48.10-65.91)	48.34 (45.50–53.90)	53.89 (46.13–74.51)	64.53 (60.12–77.10)
**Selected metabolites of the TCA cycle, median (IQR)**				
Glucose (mmol/L)	6.53 (6.10–7.19)	6.51 (6.26–7.14)	6.39 (5.43–7.72)	7.25 (6.81–7.80)
Pyruvic acid (mmol/L)	0.08 (0.07–0.12)	0.08 (0.07–0.11)	0.14 (0.06–0.14)	0.09 (0.07–0.10)
Succinic acid (mmol/L)	0.00 (0.00-0.01)	0.00 (0.00-0.01)	0.00 (0.00–0.00)	0.01 (0.00-0.02)
Lactic acid (mmol/L)	1.57 (1.23–1.91)	1.65 (1.40–1.94)	1.89 (1.42–2.06)	1.66 (1.38–1.95)

**Fig. 1:**
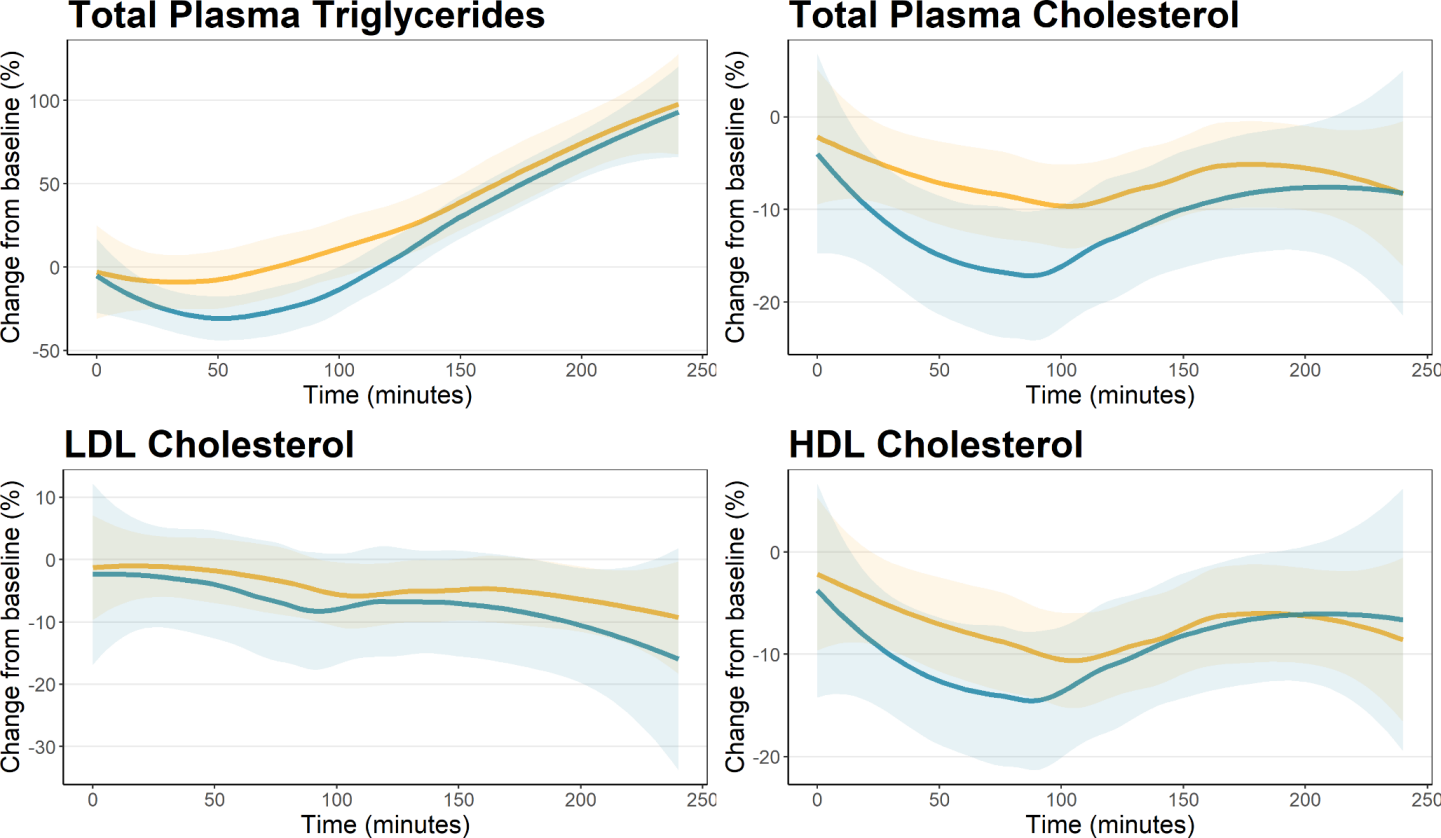
Difference between the 90 s ischemia group and control for the main lipoprotein components. Blue 90 s ischemia, yellow – control

**Fig. 2:**
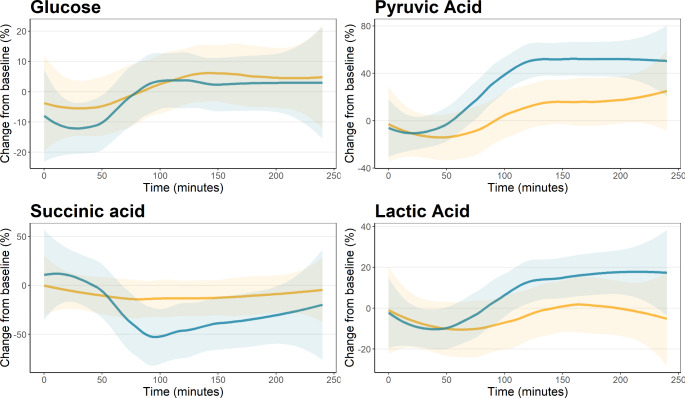
Difference between the 90 s ischemia group and control for selected metabolites of the Tricarboxylic Acid Cycle. Blue 90 s ischemia, yellow – control

Table 2 shows the metabolites with significant changes in all groups over the 4 h. Only 4 of 39 non-lipid metabolites showed a significant change, two TCA associated organic acids (pyruvic acid and acetic acid) and two amino acids (sarcosine and glutamine). However, the lipid metabolism changed substantially as 38 of 112 (34%) lipoprotein parameters showed significant changes. Changes for the 30- and 60- and 90 s ischemia groups compared to controls are shown in supplementary Tables 4–6. Figure 1 shows the relative change from baseline of the main lipoprotein components. An overall decrease in total plasma triglycerides was observed over the first hour followed by a normalization and increase. In contrast, total plasma cholesterol decreased for both groups and stayed reduced throughout the 4 h, with the 90 s group having a comparatively larger decrease during the first 2 h. There were no group differences for either total LDL or HDL cholesterol which both decreased during the period. Figure 2 shows the relative change from baseline of selected components of the TCA cycle. This showed a small decrease in plasma glucose with no differences between groups. For both pyruvic acid and lactic acid there was a small decrease in concentration over the first hour followed by a sharp increase with a significantly higher increase for the 90 s ischemia group than the control. The relative changes for the main lipoprotein components and the selected components of the TCA cycle in the 30- and 60 s ischemia groups are shown in supplementary Figs. 1–2.

**Table 2:** Results from 2-way ANOVA of each metabolite between the 30 s, 60 s, 90 s ischemia and control groups. Selected here are all metabolites that had a significant time and group difference. LDL – Low Density Lipoprotein, HDL – High Density Lipoprotein, VLDL – Very Low Density Lipoprotein, IDL - Intermediate Density Lipoprotein

Name/abbreviation of metabolite	Subclass	Compound	P for difference over time	P for difference between groups
**Non lipids**				
Glutamine	Amino acid		0.0005	0.0077
Sarcosine	Amino acid		< 0.0001	0.0170
Acetic acid	Organic acid		0.0043	0.0136
Pyruvic acid	Organic acid		< 0.0001	0.0018
**Lipoproteins**				
H4A1	HDL-4 Subclass	Apolipoprotein-A1	< 0.0001	< 0.0001
H1A2	HDL-1 Subclass	Apolipoprotein-A2	< 0.0001	0.0019
H2A2	HDL-2 Subclass	Apolipoprotein-A2	< 0.0001	0.0006
H3A2	HDL-3 Subclass	Apolipoprotein-A2	0.0048	0.0138
H4A2	HDL-4 Subclass	Apolipoprotein-A2	< 0.0001	0.0088
L1AB	LDL-1 Subclass	Apolipoprotein-B100	< 0.0001	0.0002
L2AB	LDL-2 Subclass	Apolipoprotein-B100	< 0.0001	0.0059
L3AB	LDL-3 Subclass	Apolipoprotein-B100	< 0.0001	< 0.0001
L5AB	LDL-5 Subclass	Apolipoprotein-B100	0.0010	0.0264
H4CH	HDL-4 Subclass	Cholesterol	< 0.0001	< 0.0001
LDCH	LDL	Cholesterol	0.0034	0.0001
L1CH	LDL-1 Subclass	Cholesterol	< 0.0001	< 0.0001
L2CH	LDL-2 Subclass	Cholesterol	< 0.0001	0.0122
L3CH	LDL-3 Subclass	Cholesterol	< 0.0001	< 0.0001
L5CH	LDL-5 Subclass	Cholesterol	0.0009	0.0192
V5CH	VLDL-5 Subclass	Cholesterol	0.0329	0.0116
H2FC	HDL-2 Subclass	Free Cholesterol	0.0003	0.0188
H4FC	HDL-4 Subclass	Free Cholesterol	< 0.0001	0.0047
L1FC	LDL-1 Subclass	Free Cholesterol	< 0.0001	0.0004
L3FC	LDL-3 Subclass	Free Cholesterol	< 0.0001	0.0076
L4FC	LDL-4 Subclass	Free Cholesterol	< 0.0001	< 0.0001
LDFC	LDL Class	Free Cholesterol	< 0.0001	0.0006
LDHD	Ratio LDL and HDL Cholesterol	LDL Cholesterol / HDL Cholesterol	0.0338	< 0.0001
L1PN	LDL-1	Particle Number	< 0.0001	0.0002
L2PN	LDL-2	Particle Number	< 0.0001	0.0059
L3PN	LDL-3	Particle Number	< 0.0001	< 0.0001
L5PN	LDL-5	Particle Number	0.0010	0.0264
H4PL	HDL-4 Subclass	Phospholipids	< 0.0001	0.0003
IDPL	IDL Class	Phospholipids	< 0.0001	0.0068
L1PL	LDL-1 Subclass	Phospholipids	< 0.0001	< 0.0001
L2PL	LDL-2 Subclass	Phospholipids	< 0.0001	0.0117
L3PL	LDL-3 Subclass	Phospholipids	< 0.0001	< 0.0001
L5PL	LDL-5 Subclass	Phospholipids	0.0003	0.0224
LDPL	LDL Class	Phospholipids	0.0008	0.0002
H1TG	HDL-1 Subclass	Triglycerides	< 0.0001	0.0191
H2TG	HDL-2 Subclass	Triglycerides	< 0.0001	0.0056
HDTG	HDL Class	Triglycerides	< 0.0001	0.0253
L5TG	LDL-5 Subclass	Triglycerides	< 0.0001	0.0047

**Fig. 3:**
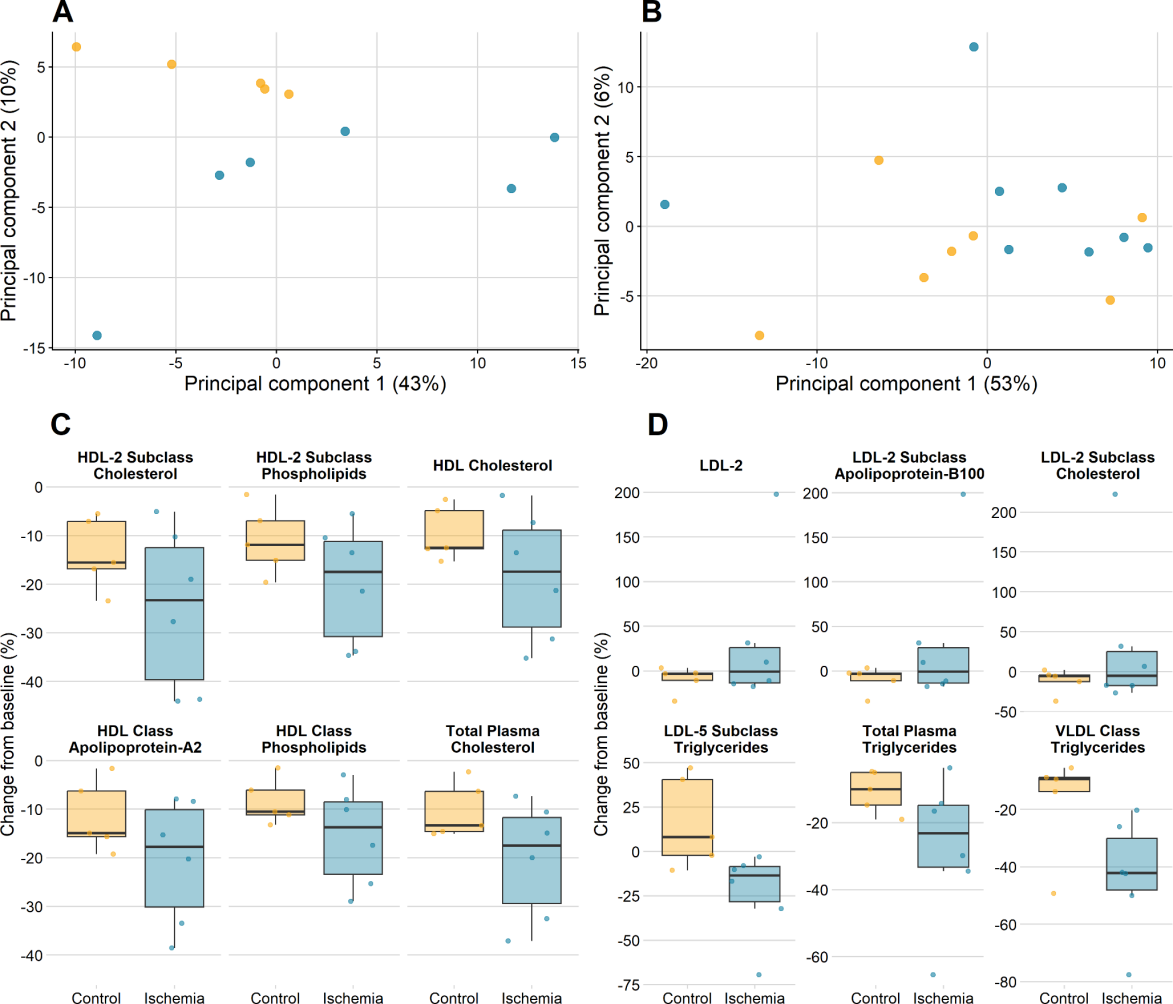
Principal component analysis of changes in metabolite concentrations and lipoprotein parameters the 90 s ischemia group and control after 15 min (A) and 60 min (B), and metabolite concentrations and lipoprotein parameters that change along PC1 (C) and PC2 (D) after 15 min. All concentration changes are displayed as percentage changefrom baseline – Control samples are shown in yellow and 90 s ischemic samples in blue,. The percentages displayed in panel A and B denote the variation explained by the principal components. LDL – Low Density Lipoprotein, HDL – High Density Lipoprotein

The PCA score plots of the concentration changes 15 and 60 min after the control treatment and 90 s ischemia are shown in Fig. 3. After 15 min principal component 1 (PC1) and 2 explain 43% and 10% of the variation, and after 60 min they explain 53% and 6%. After 15 min, we observed a significant effect of ischemia (p = 0.013) on PC 2, while no significant effects were seen on PC1 (Fig. 3A). However, we notice that the standard deviation for the ischemic samples is much larger than for the control samples for both PCs (standard deviation ratios: 2.0 and 3.8). After 60 min, none of the PCs show significant effects or changes in standard deviation (Fig. 3B). After 15 min the most important contributors to PC1 were parameters related to subclasses of HDL (5 of 6) along with total plasma cholesterol. The important contributors to PC2 were parameters associated with triglycerides and LDL. When looking at the lipoprotein parameters that showed the most important contributions to the loadings, there seemed to be a threshold effect at 90 s ischemia (supplementary Fig. 3), we compared data from the 90 s ischemia group with the pooled data from the remaining 3 groups. This showed a significant difference for all 5 HDL metabolites in the 15 min analysis with total plasma cholesterol not reaching significance (p = 0.062). In the 60 min analysis only total plasma apolipoprotein-B100 and apolipoprotein-B100 carrying particles reached significance.

## Discussion

This study is the first to describe the pattern of temporal changes in metabolites after myocardial ischemia in humans using ^1^ H NMR. This gives a unique view of the metabolic changes associated with brief myocardial ischemia over the first 4 h. We found that ischemia caused changes in a large subset of lipoprotein subclasses. Our study showed substantial changes in the metabolism as early as 15 min after brief ischemia. Especially HDL related parameters including total HDL cholesterol showed effects early after ischemia.

There are a few previous studies of metabolic changes from similar experimental setups of induced ischemia in humans (Bodi et al., 2012; Chacko et al., 2021). In both cases patients were included when undergoing PCI, which means they all had varying degrees of significant CAD. In our cohort, all patients had normal coronary arteries before inclusion. Further, in the recent study by Chacko et al. (Chacko et al., 2021), the 46 patients included underwent induced ischemia until presentation of symptoms or electrocardiographic changes or maximal duration up to 1 min. This resulted in patients receiving a variable degree of ischemia with an average of about 30 s. Thus, compared to the present study, our patients had a significantly longer period of induced ischemia. Further, the following sample intervals were very short (1 and 5 min post induced ischemia), giving the data limited usefulness in a clinical context where patients on average present about 2 h after the onset of symptoms (Ting et al., 2008).

We expected to see an increase in lactic acid as a result of the myocardial ischemia inhibiting the TCA cycle leading to an increase in anaerobic metabolism. However, this increase was relatively slow only materializing 1–2 h after the ischemia. This delay was not observed in a similar previous study, in which lactic acid was elevated as early as 20 min post induced ischemia (Bodi et al., 2012). The changes observed in the lipid metabolism could be partially explained by the rise of catecholamines caused by the pain induced by ischemia, as this is known to lead to activation of specific lipases which work in adipose tissue (Pitt et al., 2008). Though changes in the lipid metabolism is evident in this study, previous observational data of patients with ST-elevation MI showed that even if LDL changes were observed in the hours after ischemia, these changes not large enough to justify the use of LDL cholesterol to guide treatment, e.g. with statins (Pitt et al., 2008).

### Limitations

The primary limitation of our study is the relatively small sample size of each group. However, having multiple measurements for each participant makes the comparisons much stronger as studies have shown that while the inter-individual metabolome can vary wildly, the individual’s metabolome is fairly consistent even measured over days (Agueusop et al., 2020). Still, this work will require further research in larger cohorts. It may well be that the rather limited effects detected here are a consequence of a strong metabolite response to the control procedure, rather than a lack of metabolite response to myocardial ischemia.

## Conclusion

In this study we investigated the earliest temporal changes in metabolites of patients undergoing brief myocardial ischemia in an unbiased setup using ^1^ H NMR. We found that the ischemia led to changes throughout the lipid metabolism as early as 15 min after the intervention. Further, we found a surprising delay in the rise of lactic acid.

## Electronic supplementary material

Below is the link to the electronic supplementary material.

Supplementary Material 1

## Data Availability

The datasets generated during and/or analyzed during the current study are available from the corresponding author on reasonable request.
