# Supplementary material for: Metabolomics of early myocardial ischemia
Source: Metabolomics. 2023 Apr 1;19(4):33. doi: 10.1007/s11306-023-01999-8 (PMC10066099; doi:10.1007/s11306-023-01999-8)

Supplementary materials for “Metabolomics of early myocardial ischemia”

Contents

[Supplementary table 1 – Metabolites included in the principal component analysis of difference between 0 and 15 minutes between the 90 second ischemia group and control 3](#_Toc101507900)

[Supplementary table 2 – Metabolites included in the principal component analysis of difference between 0 and 60 minutes between the 90 second ischemia group and control 7](#_Toc101507901)

[Supplementary table 3 – Baseline measurements for all metabolites and lipoprotein subclasses 11](#_Toc101507902)

[Supplementary Table 4 – Results from 2-way ANOVA of each metabolite between 30 second ischemia and control groups. Selected here are all metabolites that had a significant time and group difference. LDL – Low Density Lipoprotein, HDL – High Density Lipoprotein, VLDL – Very Low Density Lipoprotein, IDL - Intermediate Density Lipoprotein 19](#_Toc101507903)

[Supplementary Table 3 – Results from 2-way ANOVA of each metabolite between 60 second ischemia and control groups. Selected here are all metabolites that had a significant time and group difference. LDL – Low Density Lipoprotein, HDL – High Density Lipoprotein, VLDL – Very Low Density Lipoprotein, IDL - Intermediate Density Lipoprotein 21](#_Toc101507904)

[Supplementary Fig.1 – Difference between the 60 second ischemia group, 30 second ischemia group and control for the main lipoprotein components. Red – 60 seconds ischemia, Blue – 30 seconds ischemia, yellow – control 23](#_Toc101507905)

[Supplementary Fig.2 – Difference between the 60 second ischemia group, 30 second ischemia group and control for selected metabolites of the Tricarboxylic Acid Cycle. Red – 60 seconds ischemia, blue – 30 seconds ischemia, yellow – control 24](#_Toc101507906)

[Supplementary Fig.3 – Quantification of selected metabolites from the principal component analyses for time 0 to 15 minutes (A) and 0 to 60 minutes (B) for all groups. Yellow – control, blue – 30 seconds ischemia, red – 60 seconds ischemia, green – 90 seconds ischemia. LDL – Low Density Lipoprotein, HDL – High Density Lipoprotein. 25](#_Toc101507907)

## Supplementary table 1 – Metabolites included in the principal component analysis of difference between 0 and 15 minutes between the 90 second ischemia group and control. LDL – Low Density Lipoprotein, HDL – High Density Lipoprotein, VLDL – Very Low Density Lipoprotein, IDL - Intermediate Density Lipoprotein

| **Metabolite** | **Subclass** | **Compound** |
| --- | --- | --- |
| TPTG | Total Plasma | Triglycerides |
| TPCH | Total Plasma | Cholesterol |
| LDCH | LDL | Cholesterol |
| HDCH | HDL | Cholesterol |
| TPA1 | Total Plasma | Apolipoprotein-A1 |
| TPA2 | Total Plasma | Apolipoprotein-A2 |
| TPAB | Total Plasma | Apolipoprotein-B100 |
| LDHD | Ratio LDL and HDL Cholesterol | LDL Cholesterol / HDL Cholesterol |
| ABA1 | Ratio of Apolipoproteins A1 and B100 | Apolipoprotein-A1 / Apolipoprotein-B100 |
| TBPN | Apolipoprotein-B100 carrying particles | Particle Number |
| VLPN | VLDL | Particle Number |
| IDPN | IDL | Particle Number |
| LDPN | LDL | Particle Number |
| L1PN | LDL-1 | Particle Number |
| L2PN | LDL-2 | Particle Number |
| L3PN | LDL-3 | Particle Number |
| L4PN | LDL-4 | Particle Number |
| L5PN | LDL-5 | Particle Number |
| L6PN | LDL-6 | Particle Number |
| VLTG | VLDL Class | Triglycerides |
| LDTG | LDL Class | Triglycerides |
| HDTG | HDL Class | Triglycerides |
| VLCH | VLDL Class | Cholesterol |
| IDCH | IDL Class | Cholesterol |
| VLFC | VLDL Class | Free Cholesterol |
| IDFC | IDL Class | Free Cholesterol |
| LDFC | LDL Class | Free Cholesterol |
| HDFC | HDL Class | Free Cholesterol |
| VLPL | VLDL Class | Phospholipids |
| IDPL | IDL Class | Phospholipids |
| LDPL | LDL Class | Phospholipids |
| HDPL | HDL Class | Phospholipids |
| HDA1 | HDL Class | Apolipoprotein-A1 |
| HDA2 | HDL Class | Apolipoprotein-A2 |
| VLAB | VLDL Class | Apolipoprotein-B100 |
| IDAB | IDL Class | Apolipoprotein-B100 |
| LDAB | LDL Class | Apolipoprotein-B100 |
| V1TG | VLDL-1 Subclass | Triglycerides |
| V2TG | VLDL-2 Subclass | Triglycerides |
| V4TG | VLDL-4 Subclass | Triglycerides |
| V5TG | VLDL-5 Subclass | Triglycerides |
| V1CH | VLDL-1 Subclass | Cholesterol |
| V2CH | VLDL-2 Subclass | Cholesterol |
| V3CH | VLDL-3 Subclass | Cholesterol |
| V4CH | VLDL-4 Subclass | Cholesterol |
| V2FC | VLDL-2 Subclass | Free Cholesterol |
| V4FC | VLDL-4 Subclass | Free Cholesterol |
| V1PL | VLDL-1 Subclass | Phospholipids |
| V2PL | VLDL-2 Subclass | Phospholipids |
| V3PL | VLDL-3 Subclass | Phospholipids |
| V4PL | VLDL-4 Subclass | Phospholipids |
| L1TG | LDL-1 Subclass | Triglycerides |
| L2TG | LDL-2 Subclass | Triglycerides |
| L3TG | LDL-3 Subclass | Triglycerides |
| L4TG | LDL-4 Subclass | Triglycerides |
| L5TG | LDL-5 Subclass | Triglycerides |
| L6TG | LDL-6 Subclass | Triglycerides |
| L1CH | LDL-1 Subclass | Cholesterol |
| L2CH | LDL-2 Subclass | Cholesterol |
| L3CH | LDL-3 Subclass | Cholesterol |
| L4CH | LDL-4 Subclass | Cholesterol |
| L5CH | LDL-5 Subclass | Cholesterol |
| L6CH | LDL-6 Subclass | Cholesterol |
| L1FC | LDL-1 Subclass | Free Cholesterol |
| L2FC | LDL-2 Subclass | Free Cholesterol |
| L3FC | LDL-3 Subclass | Free Cholesterol |
| L4FC | LDL-4 Subclass | Free Cholesterol |
| L5FC | LDL-5 Subclass | Free Cholesterol |
| L6FC | LDL-6 Subclass | Free Cholesterol |
| L1PL | LDL-1 Subclass | Phospholipids |
| L2PL | LDL-2 Subclass | Phospholipids |
| L3PL | LDL-3 Subclass | Phospholipids |
| L4PL | LDL-4 Subclass | Phospholipids |
| L5PL | LDL-5 Subclass | Phospholipids |
| L6PL | LDL-6 Subclass | Phospholipids |
| L1AB | LDL-1 Subclass | Apolipoprotein-B100 |
| L2AB | LDL-2 Subclass | Apolipoprotein-B100 |
| L3AB | LDL-3 Subclass | Apolipoprotein-B100 |
| L4AB | LDL-4 Subclass | Apolipoprotein-B100 |
| L5AB | LDL-5 Subclass | Apolipoprotein-B100 |
| L6AB | LDL-6 Subclass | Apolipoprotein-B100 |
| H1TG | HDL-1 Subclass | Triglycerides |
| H2TG | HDL-2 Subclass | Triglycerides |
| H3TG | HDL-3 Subclass | Triglycerides |
| H4TG | HDL-4 Subclass | Triglycerides |
| H1CH | HDL-1 Subclass | Cholesterol |
| H2CH | HDL-2 Subclass | Cholesterol |
| H3CH | HDL-3 Subclass | Cholesterol |
| H4CH | HDL-4 Subclass | Cholesterol |
| H1FC | HDL-1 Subclass | Free Cholesterol |
| H2FC | HDL-2 Subclass | Free Cholesterol |
| H3FC | HDL-3 Subclass | Free Cholesterol |
| H4FC | HDL-4 Subclass | Free Cholesterol |
| H1PL | HDL-1 Subclass | Phospholipids |
| H2PL | HDL-2 Subclass | Phospholipids |
| H3PL | HDL-3 Subclass | Phospholipids |
| H4PL | HDL-4 Subclass | Phospholipids |
| H1A1 | HDL-1 Subclass | Apolipoprotein-A1 |
| H2A1 | HDL-2 Subclass | Apolipoprotein-A1 |
| H3A1 | HDL-3 Subclass | Apolipoprotein-A1 |
| H4A1 | HDL-4 Subclass | Apolipoprotein-A1 |
| H1A2 | HDL-1 Subclass | Apolipoprotein-A2 |
| H2A2 | HDL-2 Subclass | Apolipoprotein-A2 |
| H3A2 | HDL-3 Subclass | Apolipoprotein-A2 |
| H4A2 | HDL-4 Subclass | Apolipoprotein-A2 |
| Alanine | Amino acid |  |
| Creatine | Amino acid |  |
| Creatinine | Amino acid |  |
| Glutamine | Amino acid |  |
| Glycine | Amino acid |  |
| Histidine | Amino acid |  |
| Isoleucine | Amino acid |  |
| Leucine | Amino acid |  |
| Methionine | Amino acid |  |
| N,N-Dimethylglycine | Amino acid |  |
| Phenylalanine | Amino acid |  |
| Sarcosine | Amino acid |  |
| Tyrosine | Amino acid |  |
| Valine | Amino acid |  |
| Acetic acid | Organic acid |  |
| Citric acid | Organic acid |  |
| Formic acid | Organic acid |  |
| Lactic acid | Organic acid |  |
| Succinic acid | Organic acid |  |
| Acetoacetic acid | Organic acid |  |
| Acetone | Other |  |
| Pyruvic acid | Organic acid |  |
| Glucose | Other |  |
| Dimethylsulfone | Other |  |

## Supplementary table 2 – Metabolites included in the principal component analysis of difference between 0 and 60 minutes between the 90 second ischemia group and control. LDL – Low Density Lipoprotein, HDL – High Density Lipoprotein, VLDL – Very Low Density Lipoprotein, IDL - Intermediate Density Lipoprotein

| **Metabolite** | **Subclass** | **Compound** |
| --- | --- | --- |
| TPTG | Total Plasma | Triglycerides |
| TPCH | Total Plasma | Cholesterol |
| LDCH | LDL | Cholesterol |
| HDCH | HDL | Cholesterol |
| TPA1 | Total Plasma | Apolipoprotein-A1 |
| TPA2 | Total Plasma | Apolipoprotein-A2 |
| TPAB | Total Plasma | Apolipoprotein-B100 |
| LDHD | Ratio LDL and HDL Cholesterol | LDL Cholesterol / HDL Cholesterol |
| ABA1 | Ratio of Apolipoproteins A1 and B100 | Apolipoprotein-A1 / Apolipoprotein-B100 |
| TBPN | Apolipoprotein-B100 carrying particles | Particle Number |
| VLPN | VLDL | Particle Number |
| IDPN | IDL | Particle Number |
| LDPN | LDL | Particle Number |
| L1PN | LDL-1 | Particle Number |
| L2PN | LDL-2 | Particle Number |
| L3PN | LDL-3 | Particle Number |
| L4PN | LDL-4 | Particle Number |
| L5PN | LDL-5 | Particle Number |
| L6PN | LDL-6 | Particle Number |
| VLTG | VLDL Class | Triglycerides |
| LDTG | LDL Class | Triglycerides |
| HDTG | HDL Class | Triglycerides |
| VLCH | VLDL Class | Cholesterol |
| IDCH | IDL Class | Cholesterol |
| VLFC | VLDL Class | Free Cholesterol |
| IDFC | IDL Class | Free Cholesterol |
| LDFC | LDL Class | Free Cholesterol |
| HDFC | HDL Class | Free Cholesterol |
| VLPL | VLDL Class | Phospholipids |
| IDPL | IDL Class | Phospholipids |
| LDPL | LDL Class | Phospholipids |
| HDPL | HDL Class | Phospholipids |
| HDA1 | HDL Class | Apolipoprotein-A1 |
| HDA2 | HDL Class | Apolipoprotein-A2 |
| VLAB | VLDL Class | Apolipoprotein-B100 |
| IDAB | IDL Class | Apolipoprotein-B100 |
| LDAB | LDL Class | Apolipoprotein-B100 |
| V1TG | VLDL-1 Subclass | Triglycerides |
| V2TG | VLDL-2 Subclass | Triglycerides |
| V3TG | VLDL-3 Subclass | Triglycerides |
| V4TG | VLDL-4 Subclass | Triglycerides |
| V5TG | VLDL-5 Subclass | Triglycerides |
| V1CH | VLDL-1 Subclass | Cholesterol |
| V3CH | VLDL-3 Subclass | Cholesterol |
| V4CH | VLDL-4 Subclass | Cholesterol |
| V3FC | VLDL-3 Subclass | Free Cholesterol |
| V4FC | VLDL-4 Subclass | Free Cholesterol |
| V1PL | VLDL-1 Subclass | Phospholipids |
| V2PL | VLDL-2 Subclass | Phospholipids |
| V3PL | VLDL-3 Subclass | Phospholipids |
| V4PL | VLDL-4 Subclass | Phospholipids |
| L1TG | LDL-1 Subclass | Triglycerides |
| L2TG | LDL-2 Subclass | Triglycerides |
| L3TG | LDL-3 Subclass | Triglycerides |
| L4TG | LDL-4 Subclass | Triglycerides |
| L5TG | LDL-5 Subclass | Triglycerides |
| L6TG | LDL-6 Subclass | Triglycerides |
| L1CH | LDL-1 Subclass | Cholesterol |
| L2CH | LDL-2 Subclass | Cholesterol |
| L3CH | LDL-3 Subclass | Cholesterol |
| L4CH | LDL-4 Subclass | Cholesterol |
| L5CH | LDL-5 Subclass | Cholesterol |
| L6CH | LDL-6 Subclass | Cholesterol |
| L1FC | LDL-1 Subclass | Free Cholesterol |
| L2FC | LDL-2 Subclass | Free Cholesterol |
| L3FC | LDL-3 Subclass | Free Cholesterol |
| L4FC | LDL-4 Subclass | Free Cholesterol |
| L5FC | LDL-5 Subclass | Free Cholesterol |
| L6FC | LDL-6 Subclass | Free Cholesterol |
| L1PL | LDL-1 Subclass | Phospholipids |
| L2PL | LDL-2 Subclass | Phospholipids |
| L3PL | LDL-3 Subclass | Phospholipids |
| L4PL | LDL-4 Subclass | Phospholipids |
| L5PL | LDL-5 Subclass | Phospholipids |
| L6PL | LDL-6 Subclass | Phospholipids |
| L1AB | LDL-1 Subclass | Apolipoprotein-B100 |
| L2AB | LDL-2 Subclass | Apolipoprotein-B100 |
| L3AB | LDL-3 Subclass | Apolipoprotein-B100 |
| L4AB | LDL-4 Subclass | Apolipoprotein-B100 |
| L5AB | LDL-5 Subclass | Apolipoprotein-B100 |
| L6AB | LDL-6 Subclass | Apolipoprotein-B100 |
| H1TG | HDL-1 Subclass | Triglycerides |
| H2TG | HDL-2 Subclass | Triglycerides |
| H3TG | HDL-3 Subclass | Triglycerides |
| H4TG | HDL-4 Subclass | Triglycerides |
| H1CH | HDL-1 Subclass | Cholesterol |
| H2CH | HDL-2 Subclass | Cholesterol |
| H3CH | HDL-3 Subclass | Cholesterol |
| H4CH | HDL-4 Subclass | Cholesterol |
| H1FC | HDL-1 Subclass | Free Cholesterol |
| H2FC | HDL-2 Subclass | Free Cholesterol |
| H3FC | HDL-3 Subclass | Free Cholesterol |
| H4FC | HDL-4 Subclass | Free Cholesterol |
| H1PL | HDL-1 Subclass | Phospholipids |
| H2PL | HDL-2 Subclass | Phospholipids |
| H3PL | HDL-3 Subclass | Phospholipids |
| H4PL | HDL-4 Subclass | Phospholipids |
| H1A1 | HDL-1 Subclass | Apolipoprotein-A1 |
| H2A1 | HDL-2 Subclass | Apolipoprotein-A1 |
| H3A1 | HDL-3 Subclass | Apolipoprotein-A1 |
| H4A1 | HDL-4 Subclass | Apolipoprotein-A1 |
| H1A2 | HDL-1 Subclass | Apolipoprotein-A2 |
| H2A2 | HDL-2 Subclass | Apolipoprotein-A2 |
| H3A2 | HDL-3 Subclass | Apolipoprotein-A2 |
| H4A2 | HDL-4 Subclass | Apolipoprotein-A2 |
| Alanine | Amino acid |  |
| Creatine | Amino acid |  |
| Creatinine | Amino acid |  |
| Glutamine | Amino acid |  |
| Glycine | Amino acid |  |
| Histidine | Amino acid |  |
| Isoleucine | Amino acid |  |
| Leucine | Amino acid |  |
| Methionine | Amino acid |  |
| N,N-Dimethylglycine | Amino acid |  |
| Phenylalanine | Amino acid |  |
| Sarcosine | Amino acid |  |
| Tyrosine | Amino acid |  |
| Valine | Amino acid |  |
| Acetic acid | Organic acid |  |
| Citric acid | Organic acid |  |
| Lactic acid | Organic acid |  |
| Succinic acid | Organic acid |  |
| Acetoacetic acid | Organic acid |  |
| Acetone | Other |  |
| Pyruvic acid | Organic acid |  |
| Glucose | Other |  |
| Dimethylsulfone | Other |  |

## Supplementary table 3 – Baseline measurements for all metabolites and lipoprotein subclasses

|  | 0 s ischemia | 30 s ischemia | 60 s ischemia | 90 s ischemia | p |
| --- | --- | --- | --- | --- | --- |
| **n** | 9 | 8 | 9 | 8 |  |
| **TPA1 [mg/dL] (median [IQR])** | 140.28 [125.93, 162.58] | 147.04 [135.07, 150.56] | 147.02 [135.48, 174.10] | 157.81 [148.90, 182.86] | 0.473 |
| **TPA2 [mg/dL] (median [IQR])** | 31.54 [26.83, 33.56] | 34.91 [30.43, 35.97] | 35.20 [33.40, 36.45] | 34.92 [30.70, 40.48] | 0.710 |
| **TPAB [mg/dL] (median [IQR])** | 81.30 [69.32, 92.80] | 79.53 [70.45, 97.20] | 80.13 [66.67, 92.54] | 77.58 [73.27, 86.39] | 0.976 |
| **LDHD [-/-] (median [IQR])** | 1.75 [1.68, 2.35] | 2.09 [1.67, 2.39] | 1.70 [1.14, 1.95] | 1.67 [1.52, 1.95] | 0.396 |
| **ABA1 [-/-] (median [IQR])** | 0.52 [0.50, 0.66] | 0.56 [0.53, 0.64] | 0.49 [0.41, 0.61] | 0.52 [0.45, 0.56] | 0.655 |
| **TBPN [nmol/L] (median [IQR])** | 1478.26 [1260.42, 1687.37] | 1445.91 [1280.97, 1767.32] | 1457.05 [1212.28, 1682.62] | 1410.59 [1332.32, 1570.82] | 0.976 |
| **VLPN [nmol/L] (median [IQR])** | 79.11 [67.97, 103.05] | 101.19 [71.13, 140.80] | 114.67 [86.91, 179.22] | 71.76 [56.45, 107.81] | 0.271 |
| **IDPN [nmol/L] (median [IQR])** | 74.42 [56.12, 100.24] | 80.53 [68.85, 120.63] | 104.84 [97.97, 112.09] | 78.98 [60.54, 108.39] | 0.333 |
| **LDPN [nmol/L] (median [IQR])** | 1210.58 [1057.50, 1360.43] | 1211.55 [974.16, 1403.67] | 1071.81 [849.02, 1260.66] | 1169.28 [1004.50, 1349.71] | 0.819 |
| **L1PN [nmol/L] (median [IQR])** | 198.92 [180.27, 336.48] | 203.02 [160.22, 276.08] | 242.34 [152.51, 287.92] | 202.74 [168.46, 248.83] | 0.887 |
| **L2PN [nmol/L] (median [IQR])** | 197.78 [144.80, 221.99] | 195.00 [168.56, 228.48] | 149.66 [85.40, 204.33] | 153.68 [134.30, 177.47] | 0.348 |
| **L3PN [nmol/L] (median [IQR])** | 223.09 [182.92, 264.12] | 222.26 [172.78, 242.32] | 166.44 [112.75, 192.14] | 179.82 [158.38, 207.78] | 0.220 |
| **L4PN [nmol/L] (median [IQR])** | 186.43 [158.48, 201.42] | 151.11 [117.70, 205.45] | 145.42 [83.70, 172.16] | 152.55 [126.22, 208.94] | 0.423 |
| **L5PN [nmol/L] (median [IQR])** | 134.86 [89.45, 148.84] | 132.35 [128.58, 176.58] | 157.20 [90.85, 170.15] | 203.33 [145.55, 230.17] | 0.405 |
| **L6PN [nmol/L] (median [IQR])** | 229.87 [177.95, 280.13] | 259.16 [225.04, 311.91] | 309.64 [279.28, 361.28] | 328.83 [292.01, 353.62] | 0.393 |
| **VLTG [mg/dL] (median [IQR])** | 25.50 [22.57, 35.76] | 43.36 [25.66, 53.06] | 47.00 [31.79, 101.34] | 32.33 [26.87, 37.68] | 0.340 |
| **IDTG [mg/dL] (median [IQR])** | 1.07 [0.00, 1.71] | 3.22 [0.26, 5.64] | 3.27 [2.09, 11.42] | 1.19 [0.38, 2.35] | 0.162 |
| **LDTG [mg/dL] (median [IQR])** | 16.46 [15.93, 20.79] | 17.67 [14.52, 21.52] | 18.44 [15.43, 20.48] | 19.80 [16.90, 23.59] | 0.783 |
| **HDTG [mg/dL] (median [IQR])** | 7.56 [7.11, 9.30] | 7.89 [7.56, 9.51] | 9.19 [8.37, 10.97] | 8.39 [7.33, 10.07] | 0.382 |
| **VLCH [mg/dL] (median [IQR])** | 13.37 [8.64, 13.71] | 17.76 [8.86, 27.43] | 17.43 [13.55, 27.93] | 8.04 [6.45, 16.55] | 0.161 |
| **IDCH [mg/dL] (median [IQR])** | 10.26 [6.73, 13.83] | 13.40 [11.16, 16.91] | 16.22 [12.33, 16.36] | 9.34 [8.27, 16.59] | 0.411 |
| **VLFC [mg/dL] (median [IQR])** | 5.48 [4.20, 6.01] | 6.73 [4.44, 10.11] | 7.20 [5.97, 11.76] | 4.17 [3.68, 7.37] | 0.302 |
| **IDFC [mg/dL] (median [IQR])** | 2.34 [1.80, 3.83] | 3.45 [2.83, 4.51] | 4.15 [3.31, 4.59] | 2.38 [1.99, 4.34] | 0.309 |
| **LDFC [mg/dL] (median [IQR])** | 35.97 [30.56, 39.34] | 34.59 [30.34, 39.20] | 28.90 [25.09, 35.10] | 33.84 [30.92, 38.45] | 0.514 |
| **HDFC [mg/dL] (median [IQR])** | 16.03 [14.31, 19.13] | 15.80 [14.38, 17.20] | 14.86 [12.76, 19.34] | 17.80 [15.23, 18.94] | 0.833 |
| **VLPL [mg/dL] (median [IQR])** | 8.38 [7.96, 10.66] | 12.08 [6.26, 17.29] | 14.26 [7.90, 26.19] | 8.16 [6.41, 13.99] | 0.490 |
| **IDPL [mg/dL] (median [IQR])** | 3.36 [2.50, 4.36] | 4.76 [3.79, 5.75] | 6.02 [3.43, 6.71] | 3.76 [2.88, 6.64] | 0.415 |
| **LDPL [mg/dL] (median [IQR])** | 61.99 [54.50, 70.30] | 60.64 [55.11, 71.49] | 55.28 [48.72, 61.03] | 59.73 [54.09, 66.18] | 0.632 |
| **HDPL [mg/dL] (median [IQR])** | 73.22 [68.52, 97.62] | 79.41 [69.38, 86.34] | 83.15 [65.92, 98.16] | 85.82 [78.13, 96.30] | 0.755 |
| **HDA1 [mg/dL] (median [IQR])** | 137.33 [121.25, 165.51] | 144.10 [131.37, 149.46] | 143.80 [130.52, 175.61] | 157.37 [146.70, 184.54] | 0.504 |
| **HDA2 [mg/dL] (median [IQR])** | 31.68 [27.70, 32.31] | 34.37 [29.64, 35.22] | 34.84 [33.02, 35.95] | 34.63 [30.91, 40.25] | 0.626 |
| **VLAB [mg/dL] (median [IQR])** | 4.35 [3.74, 5.67] | 5.56 [3.91, 7.74] | 6.31 [4.78, 9.86] | 3.95 [3.10, 5.93] | 0.265 |
| **IDAB [mg/dL] (median [IQR])** | 4.09 [3.09, 5.51] | 4.43 [3.78, 6.64] | 5.77 [5.39, 6.16] | 4.35 [3.33, 5.96] | 0.333 |
| **LDAB [mg/dL] (median [IQR])** | 66.58 [58.16, 74.82] | 66.63 [53.58, 77.19] | 58.95 [46.69, 69.33] | 64.31 [55.24, 74.23] | 0.819 |
| **V1TG [mg/dL] (median [IQR])** | 12.87 [5.46, 16.57] | 14.05 [7.77, 22.53] | 15.08 [12.44, 63.55] | 9.59 [6.06, 13.43] | 0.560 |
| **V2TG [mg/dL] (median [IQR])** | 3.17 [2.02, 6.87] | 7.34 [4.56, 10.15] | 6.67 [4.67, 11.31] | 5.40 [4.36, 6.41] | 0.215 |
| **V3TG [mg/dL] (median [IQR])** | 4.63 [2.35, 5.27] | 8.56 [4.70, 12.52] | 7.85 [6.16, 11.03] | 5.47 [4.48, 7.20] | 0.096 |
| **V4TG [mg/dL] (median [IQR])** | 5.95 [3.03, 7.32] | 6.88 [5.81, 9.30] | 9.36 [6.12, 10.06] | 5.85 [4.96, 7.74] | 0.102 |
| **V5TG [mg/dL] (median [IQR])** | 3.32 [3.01, 3.44] | 2.90 [2.54, 3.56] | 3.46 [3.09, 3.63] | 2.52 [2.31, 3.76] | 0.635 |
| **V1CH [mg/dL] (median [IQR])** | 2.91 [2.68, 3.81] | 6.05 [2.06, 8.12] | 4.56 [2.44, 12.51] | 1.51 [0.79, 4.07] | 0.207 |
| **V2CH [mg/dL] (median [IQR])** | 0.91 [0.40, 1.54] | 1.80 [0.87, 3.43] | 1.52 [1.10, 2.46] | 0.90 [0.61, 1.67] | 0.426 |
| **V3CH [mg/dL] (median [IQR])** | 1.76 [1.02, 2.58] | 3.87 [2.13, 6.23] | 3.35 [2.76, 4.89] | 1.85 [1.81, 2.29] | 0.083 |
| **V4CH [mg/dL] (median [IQR])** | 2.94 [2.51, 3.81] | 3.86 [3.12, 6.38] | 4.94 [4.38, 5.61] | 3.12 [2.58, 4.11] | 0.295 |
| **V5CH [mg/dL] (median [IQR])** | 1.26 [1.03, 1.67] | 1.16 [0.75, 1.50] | 1.20 [0.92, 1.37] | 0.46 [0.03, 1.79] | 0.818 |
| **V1FC [mg/dL] (median [IQR])** | 0.00 [0.00, 0.45] | 0.48 [0.00, 1.07] | 0.71 [0.00, 3.83] | 0.05 [0.00, 0.52] | 0.404 |
| **V2FC [mg/dL] (median [IQR])** | 0.27 [0.07, 0.56] | 1.03 [0.26, 1.56] | 0.67 [0.34, 1.63] | 0.25 [0.11, 0.55] | 0.273 |
| **V3FC [mg/dL] (median [IQR])** | 0.75 [0.27, 0.78] | 1.35 [0.62, 2.14] | 1.29 [0.77, 2.27] | 0.58 [0.44, 0.76] | 0.120 |
| **V4FC [mg/dL] (median [IQR])** | 1.49 [1.34, 1.88] | 2.02 [1.75, 3.48] | 2.80 [2.03, 3.13] | 1.23 [0.96, 1.99] | 0.108 |
| **V5FC [mg/dL] (median [IQR])** | 0.59 [0.42, 0.80] | 0.71 [0.35, 1.04] | 0.74 [0.58, 1.08] | 0.14 [0.00, 0.76] | 0.286 |
| **V1PL [mg/dL] (median [IQR])** | 2.42 [1.40, 3.11] | 3.96 [1.94, 5.18] | 3.14 [1.77, 10.77] | 1.73 [1.46, 2.67] | 0.313 |
| **V2PL [mg/dL] (median [IQR])** | 0.84 [0.40, 1.71] | 1.98 [1.16, 2.88] | 1.79 [0.98, 3.00] | 1.29 [1.08, 1.46] | 0.279 |
| **V3PL [mg/dL] (median [IQR])** | 1.60 [0.88, 1.82] | 2.59 [1.37, 3.65] | 2.93 [1.41, 4.03] | 1.50 [1.26, 1.90] | 0.209 |
| **V4PL [mg/dL] (median [IQR])** | 3.35 [2.49, 3.78] | 3.96 [3.32, 5.69] | 5.21 [3.80, 5.29] | 3.08 [2.65, 3.83] | 0.130 |
| **V5PL [mg/dL] (median [IQR])** | 1.49 [1.26, 1.89] | 1.59 [1.10, 2.11] | 1.73 [1.60, 1.85] | 0.75 [0.47, 2.28] | 0.829 |
| **L1TG [mg/dL] (median [IQR])** | 4.41 [3.49, 5.33] | 4.89 [4.21, 5.76] | 5.81 [4.91, 6.79] | 5.24 [4.29, 6.86] | 0.506 |
| **L2TG [mg/dL] (median [IQR])** | 1.62 [1.54, 2.80] | 1.58 [1.19, 2.20] | 2.29 [1.38, 2.35] | 1.86 [1.48, 2.80] | 0.594 |
| **L3TG [mg/dL] (median [IQR])** | 2.65 [2.31, 2.87] | 2.56 [1.74, 2.63] | 2.00 [1.68, 2.78] | 2.18 [1.85, 3.05] | 0.444 |
| **L4TG [mg/dL] (median [IQR])** | 1.88 [1.61, 2.18] | 1.69 [0.79, 2.25] | 1.45 [0.99, 1.56] | 1.96 [1.56, 2.79] | 0.236 |
| **L5TG [mg/dL] (median [IQR])** | 1.59 [1.06, 1.78] | 1.40 [0.81, 1.98] | 1.63 [1.20, 1.83] | 2.32 [1.61, 2.53] | 0.205 |
| **L6TG [mg/dL] (median [IQR])** | 3.70 [2.83, 4.65] | 3.82 [3.69, 4.53] | 4.51 [3.96, 5.04] | 4.64 [4.33, 5.57] | 0.314 |
| **L1CH [mg/dL] (median [IQR])** | 21.76 [18.99, 34.94] | 20.08 [16.93, 27.85] | 24.02 [14.68, 28.93] | 19.58 [16.45, 23.83] | 0.834 |
| **L2CH [mg/dL] (median [IQR])** | 20.92 [14.42, 24.70] | 19.06 [17.09, 22.56] | 13.91 [9.08, 20.79] | 15.40 [13.31, 18.33] | 0.315 |
| **L3CH [mg/dL] (median [IQR])** | 20.39 [17.43, 25.69] | 21.02 [14.66, 23.56] | 14.76 [10.05, 15.56] | 16.78 [14.85, 19.16] | 0.165 |
| **L4CH [mg/dL] (median [IQR])** | 17.20 [13.87, 17.79] | 14.68 [10.08, 18.20] | 12.19 [6.18, 12.97] | 13.53 [11.07, 18.30] | 0.448 |
| **L5CH [mg/dL] (median [IQR])** | 10.90 [6.80, 12.31] | 10.73 [9.65, 13.16] | 12.57 [5.36, 13.24] | 16.16 [10.28, 18.34] | 0.420 |
| **L6CH [mg/dL] (median [IQR])** | 13.55 [12.32, 17.12] | 15.12 [12.04, 19.48] | 21.40 [17.59, 23.18] | 21.76 [20.12, 23.28] | 0.243 |
| **L1FC [mg/dL] (median [IQR])** | 7.18 [6.26, 10.55] | 6.64 [6.10, 9.51] | 7.35 [4.91, 9.42] | 6.71 [6.22, 7.99] | 0.973 |
| **L2FC [mg/dL] (median [IQR])** | 7.06 [5.57, 8.10] | 6.57 [5.65, 8.29] | 5.48 [3.86, 7.38] | 6.82 [4.82, 7.15] | 0.721 |
| **L3FC [mg/dL] (median [IQR])** | 6.79 [6.42, 8.36] | 6.74 [5.95, 7.39] | 5.83 [3.56, 6.31] | 7.07 [5.86, 7.44] | 0.344 |
| **L4FC [mg/dL] (median [IQR])** | 5.50 [4.90, 6.55] | 5.24 [4.71, 6.44] | 4.14 [3.62, 5.06] | 5.36 [5.01, 6.52] | 0.210 |
| **L5FC [mg/dL] (median [IQR])** | 3.67 [3.17, 4.62] | 4.28 [3.96, 5.00] | 3.76 [2.98, 3.99] | 5.46 [4.40, 6.32] | 0.121 |
| **L6FC [mg/dL] (median [IQR])** | 4.32 [3.47, 5.90] | 4.84 [4.21, 5.82] | 5.09 [4.20, 6.66] | 6.51 [6.08, 7.02] | 0.267 |
| **L1PL [mg/dL] (median [IQR])** | 12.17 [10.94, 19.59] | 11.93 [10.50, 16.46] | 13.50 [9.42, 16.74] | 12.16 [9.96, 14.19] | 0.912 |
| **L2PL [mg/dL] (median [IQR])** | 11.59 [8.70, 14.03] | 10.95 [9.87, 12.76] | 8.87 [5.64, 11.91] | 9.35 [7.96, 10.52] | 0.316 |
| **L3PL [mg/dL] (median [IQR])** | 11.41 [9.78, 14.29] | 11.80 [9.00, 13.13] | 8.10 [6.13, 10.01] | 9.86 [8.60, 10.96] | 0.192 |
| **L4PL [mg/dL] (median [IQR])** | 9.59 [8.27, 9.96] | 8.11 [6.18, 10.23] | 6.87 [4.41, 7.89] | 7.93 [6.64, 10.12] | 0.439 |
| **L5PL [mg/dL] (median [IQR])** | 6.21 [4.40, 7.16] | 6.11 [5.86, 7.54] | 6.74 [4.05, 7.37] | 8.88 [6.14, 9.94] | 0.393 |
| **L6PL [mg/dL] (median [IQR])** | 8.91 [7.50, 11.15] | 9.29 [8.27, 11.54] | 13.00 [10.48, 13.39] | 12.96 [11.93, 13.77] | 0.270 |
| **L1AB [mg/dL] (median [IQR])** | 10.94 [9.91, 18.51] | 11.16 [8.81, 15.19] | 13.33 [8.39, 15.84] | 11.15 [9.27, 13.68] | 0.887 |
| **L2AB [mg/dL] (median [IQR])** | 10.88 [7.96, 12.21] | 10.73 [9.27, 12.57] | 8.23 [4.70, 11.24] | 8.46 [7.38, 9.76] | 0.348 |
| **L3AB [mg/dL] (median [IQR])** | 12.27 [10.06, 14.53] | 12.22 [9.50, 13.33] | 9.15 [6.20, 10.57] | 9.88 [8.71, 11.43] | 0.219 |
| **L4AB [mg/dL] (median [IQR])** | 10.25 [8.72, 11.08] | 8.31 [6.47, 11.30] | 8.00 [4.60, 9.47] | 8.39 [6.94, 11.49] | 0.423 |
| **L5AB [mg/dL] (median [IQR])** | 7.42 [4.92, 8.19] | 7.28 [7.07, 9.71] | 8.65 [5.00, 9.36] | 11.18 [8.01, 12.66] | 0.394 |
| **L6AB [mg/dL] (median [IQR])** | 12.64 [9.79, 15.41] | 14.25 [12.38, 17.15] | 17.03 [15.36, 19.87] | 18.09 [16.06, 19.45] | 0.393 |
| **H1TG [mg/dL] (median [IQR])** | 1.96 [1.83, 4.27] | 1.79 [1.34, 2.28] | 3.01 [2.15, 3.43] | 3.16 [1.99, 3.52] | 0.177 |
| **H2TG [mg/dL] (median [IQR])** | 1.39 [1.14, 1.72] | 1.48 [1.29, 1.68] | 1.62 [1.43, 2.07] | 1.27 [1.05, 1.62] | 0.284 |
| **H3TG [mg/dL] (median [IQR])** | 1.31 [1.25, 1.86] | 1.57 [1.21, 2.00] | 1.93 [1.64, 2.35] | 1.36 [1.14, 1.89] | 0.395 |
| **H4TG [mg/dL] (median [IQR])** | 2.16 [1.85, 2.74] | 3.03 [2.32, 3.84] | 3.20 [2.51, 3.91] | 2.70 [2.45, 2.91] | 0.206 |
| **H1CH [mg/dL] (median [IQR])** | 14.99 [13.39, 22.09] | 8.91 [5.57, 16.52] | 12.69 [11.43, 26.66] | 23.28 [15.10, 24.98] | 0.171 |
| **H2CH [mg/dL] (median [IQR])** | 9.00 [7.17, 11.80] | 9.06 [7.52, 10.44] | 9.52 [7.16, 13.10] | 10.05 [9.08, 11.45] | 0.786 |
| **H3CH [mg/dL] (median [IQR])** | 11.74 [9.84, 13.43] | 12.71 [10.68, 13.33] | 13.03 [10.25, 14.48] | 11.74 [11.27, 13.36] | 0.871 |
| **H4CH [mg/dL] (median [IQR])** | 19.13 [17.44, 20.82] | 21.02 [19.88, 22.19] | 19.95 [18.14, 21.39] | 24.07 [19.21, 26.49] | 0.293 |
| **H1FC [mg/dL] (median [IQR])** | 5.54 [4.08, 5.76] | 4.12 [3.84, 4.70] | 3.76 [3.55, 7.29] | 6.12 [5.14, 7.24] | 0.533 |
| **H2FC [mg/dL] (median [IQR])** | 2.74 [2.17, 3.60] | 3.06 [2.35, 3.16] | 2.77 [2.08, 3.52] | 2.66 [2.53, 3.27] | 0.996 |
| **H3FC [mg/dL] (median [IQR])** | 2.18 [1.85, 2.93] | 2.42 [2.02, 2.92] | 2.74 [2.12, 2.82] | 2.58 [2.38, 3.13] | 0.837 |
| **H4FC [mg/dL] (median [IQR])** | 3.98 [3.54, 5.56] | 4.83 [4.36, 5.68] | 4.33 [3.60, 5.12] | 5.43 [4.44, 6.01] | 0.261 |
| **H1PL [mg/dL] (median [IQR])** | 19.28 [17.40, 27.76] | 12.14 [11.30, 19.20] | 18.25 [14.63, 32.42] | 26.77 [16.69, 29.81] | 0.166 |
| **H2PL [mg/dL] (median [IQR])** | 13.50 [11.50, 17.88] | 14.44 [11.58, 16.37] | 15.56 [11.47, 18.59] | 14.88 [12.55, 15.46] | 0.860 |
| **H3PL [mg/dL] (median [IQR])** | 17.07 [13.55, 18.98] | 17.94 [15.74, 18.73] | 19.60 [16.34, 19.97] | 17.16 [16.07, 18.44] | 0.695 |
| **H4PL [mg/dL] (median [IQR])** | 25.30 [23.82, 27.20] | 29.00 [26.88, 31.95] | 27.22 [25.70, 29.25] | 31.08 [26.71, 31.74] | 0.351 |
| **H1A1 [mg/dL] (median [IQR])** | 19.78 [17.71, 32.65] | 10.14 [8.02, 21.58] | 18.89 [12.84, 34.82] | 31.34 [19.19, 35.56] | 0.294 |
| **H2A1 [mg/dL] (median [IQR])** | 16.66 [16.04, 22.59] | 17.90 [15.82, 19.62] | 19.33 [17.57, 21.88] | 19.92 [17.21, 22.59] | 0.712 |
| **H3A1 [mg/dL] (median [IQR])** | 28.30 [24.30, 33.90] | 31.38 [28.43, 34.03] | 32.30 [31.37, 34.14] | 29.13 [28.33, 31.36] | 0.562 |
| **H4A1 [mg/dL] (median [IQR])** | 70.22 [65.68, 74.91] | 81.62 [72.69, 82.84] | 73.38 [71.79, 79.30] | 89.16 [71.86, 93.65] | 0.267 |
| **H1A2 [mg/dL] (median [IQR])** | 2.21 [1.75, 2.96] | 1.68 [1.29, 2.24] | 2.13 [1.50, 3.17] | 2.66 [1.36, 3.60] | 0.525 |
| **H2A2 [mg/dL] (median [IQR])** | 3.50 [2.38, 3.58] | 3.10 [2.13, 3.58] | 3.86 [3.11, 4.47] | 3.30 [2.69, 3.57] | 0.368 |
| **H3A2 [mg/dL] (median [IQR])** | 6.47 [4.81, 6.57] | 6.41 [5.33, 7.05] | 7.15 [6.16, 7.73] | 6.45 [5.91, 6.82] | 0.607 |
| **H4A2 [mg/dL] (median [IQR])** | 16.94 [16.30, 19.09] | 20.11 [18.85, 21.58] | 19.75 [18.23, 20.49] | 22.54 [17.32, 23.79] | 0.442 |
| **Trimethylamine-N-oxide [mmol/L] (median [IQR])** | 0.02 [0.00, 0.02] | 0.01 [0.00, 0.02] | 0.01 [0.01, 0.02] | 0.01 [0.01, 0.02] | 0.810 |
| **Alanine [mmol/L] (median [IQR])** | 0.34 [0.33, 0.49] | 0.36 [0.31, 0.48] | 0.43 [0.28, 0.45] | 0.35 [0.32, 0.39] | 0.982 |
| **Creatine [mmol/L] (median [IQR])** | 0.02 [0.02, 0.04] | 0.03 [0.01, 0.04] | 0.02 [0.02, 0.02] | 0.02 [0.02, 0.04] | 0.778 |
| **Creatinine [mmol/L] (median [IQR])** | 0.08 [0.07, 0.09] | 0.08 [0.08, 0.10] | 0.08 [0.06, 0.09] | 0.08 [0.07, 0.10] | 0.843 |
| **Glutamine [mmol/L] (median [IQR])** | 0.66 [0.55, 0.70] | 0.49 [0.42, 0.60] | 0.54 [0.52, 0.66] | 0.72 [0.55, 0.75] | 0.090 |
| **Glycine [mmol/L] (median [IQR])** | 0.41 [0.31, 0.42] | 0.43 [0.41, 0.50] | 0.39 [0.37, 0.47] | 0.31 [0.27, 0.46] | 0.550 |
| **Histidine [mmol/L] (median [IQR])** | 0.10 [0.08, 0.11] | 0.14 [0.11, 0.14] | 0.11 [0.10, 0.14] | 0.09 [0.06, 0.12] | 0.130 |
| **Isoleucine [mmol/L] (median [IQR])** | 0.06 [0.05, 0.07] | 0.06 [0.04, 0.07] | 0.04 [0.04, 0.06] | 0.05 [0.05, 0.05] | 0.685 |
| **Leucine [mmol/L] (median [IQR])** | 0.12 [0.10, 0.13] | 0.10 [0.09, 0.12] | 0.11 [0.10, 0.14] | 0.10 [0.09, 0.11] | 0.503 |
| **Lysine [mmol/L] (median [IQR])** | 0.16 [0.11, 0.19] | 0.14 [0.11, 0.17] | 0.16 [0.12, 0.16] | 0.15 [0.10, 0.15] | 0.705 |
| **Methionine [mmol/L] (median [IQR])** | 0.07 [0.06, 0.09] | 0.05 [0.04, 0.07] | 0.07 [0.04, 0.08] | 0.07 [0.06, 0.07] | 0.246 |
| **N,N-Dimethylglycine [mmol/L] (median [IQR])** | 0.00 [0.00, 0.00] | 0.00 [0.00, 0.01] | 0.00 [0.00, 0.00] | 0.00 [0.00, 0.01] | 0.663 |
| **Phenylalanine [mmol/L] (median [IQR])** | 0.04 [0.04, 0.05] | 0.05 [0.04, 0.05] | 0.05 [0.03, 0.06] | 0.04 [0.04, 0.04] | 0.618 |
| **Sarcosine [mmol/L] (median [IQR])** | 0.00 [0.00, 0.00] | 0.00 [0.00, 0.00] | 0.00 [0.00, 0.00] | 0.00 [0.00, 0.00] | 0.229 |
| **Threonine [mmol/L] (median [IQR])** | 0.12 [0.06, 0.15] | 0.08 [0.08, 0.09] | 0.07 [0.05, 0.14] | 0.10 [0.06, 0.13] | 0.961 |
| **Tyrosine [mmol/L] (median [IQR])** | 0.05 [0.05, 0.07] | 0.05 [0.05, 0.05] | 0.06 [0.04, 0.06] | 0.05 [0.04, 0.06] | 0.562 |
| **Valine [mmol/L] (median [IQR])** | 0.29 [0.25, 0.30] | 0.29 [0.24, 0.33] | 0.27 [0.24, 0.30] | 0.24 [0.22, 0.27] | 0.449 |
| **Acetic acid [mmol/L] (median [IQR])** | 0.06 [0.05, 0.07] | 0.05 [0.04, 0.07] | 0.05 [0.04, 0.09] | 0.09 [0.08, 0.10] | 0.173 |
| **Citric acid [mmol/L] (median [IQR])** | 0.14 [0.13, 0.15] | 0.16 [0.14, 0.18] | 0.15 [0.14, 0.18] | 0.15 [0.14, 0.18] | 0.721 |
| **Formic acid [mmol/L] (median [IQR])** | 0.06 [0.05, 0.07] | 0.08 [0.05, 0.09] | 0.05 [0.04, 0.06] | 0.05 [0.04, 0.06] | 0.477 |
| **Succinic acid [mmol/L] (median [IQR])** | 0.00 [0.00, 0.01] | 0.00 [0.00, 0.01] | 0.00 [0.00, 0.00] | 0.01 [0.00, 0.02] | 0.146 |
| **3-Hydroxybutyric acid [mmol/L] (median [IQR])** | 0.07 [0.01, 0.25] | 0.09 [0.03, 0.21] | 0.07 [0.04, 0.07] | 0.15 [0.04, 0.31] | 0.691 |
| **Acetoacetic acid [mmol/L] (median [IQR])** | 0.13 [0.06, 0.13] | 0.07 [0.07, 0.12] | 0.08 [0.06, 0.10] | 0.06 [0.05, 0.09] | 0.346 |
| **Acetone [mmol/L] (median [IQR])** | 0.06 [0.04, 0.10] | 0.05 [0.03, 0.11] | 0.04 [0.03, 0.05] | 0.09 [0.06, 0.12] | 0.284 |
| **Pyruvic acid [mmol/L] (median [IQR])** | 0.08 [0.07, 0.12] | 0.08 [0.07, 0.11] | 0.14 [0.06, 0.14] | 0.09 [0.07, 0.10] | 0.835 |
| **Dimethylsulfone [mmol/L] (median [IQR])** | 0.00 [0.00, 0.01] | 0.00 [0.00, 0.00] | 0.00 [0.00, 0.00] | 0.01 [0.00, 0.01] | 0.118 |

## Supplementary table 4 – Results from 2-way ANOVA of each metabolite between 90 second ischemia and control groups. Selected here are all metabolites that had a significant time and group difference. LDL – Low Density Lipoprotein, HDL – High Density Lipoprotein, VLDL – Very Low Density Lipoprotein, IDL - Intermediate Density Lipoprotein

| **Name/abbreviation of metabolite** | **Subclass** | **Compound** | **P for difference over time** | **P for difference between groups** |
| --- | --- | --- | --- | --- |
| **Non-lipids** |  |  |  |  |
| Sarcosine | Amino acid |  | 0.0009 | 0.0045 |
| Lactic acid | Organic Acid |  | 0.0469 | 0.0341 |
| Pyruvic acid | Organic Acid |  | <0.0001 | 0.0003 |
| **Lipoproteins** |  |  |  |  |
| H4A1 | HDL-4 Subclass | Apolipoprotein-A1 | 0.0004 | <0.0001 |
| H1A2 | HDL-1 Subclass | Apolipoprotein-A2 | 0.0022 | 0.0001 |
| H2A2 | HDL-2 Subclass | Apolipoprotein-A2 | 0.0013 | <0.0001 |
| H3A2 | HDL-3 Subclass | Apolipoprotein-A2 | 0.0458 | 0.0021 |
| H4A2 | HDL-4 Subclass | Apolipoprotein-A2 | 0.0048 | 0.0060 |
| L1AB | LDL-1 Subclass | Apolipoprotein-B100 | <0.0001 | <0.0001 |
| L2AB | LDL-2 Subclass | Apolipoprotein-B100 | <0.0001 | 0.0016 |
| L3AB | LDL-3 Subclass | Apolipoprotein-B100 | <0.0001 | <0.0001 |
| VLAB | VLDL Class | Apolipoprotein-B100 | <0.0001 | 0.0292 |
| H4CH | HDL-4 Subclass | Cholesterol | 0.0003 | <0.0001 |
| LDCH | LDL | Cholesterol | 0.0065 | 0.0018 |
| L1CH | LDL-1 Subclass | Cholesterol | <0.0001 | <0.0001 |
| L2CH | LDL-2 Subclass | Cholesterol | <0.0001 | 0.0056 |
| L3CH | LDL-3 Subclass | Cholesterol | <0.0001 | <0.0001 |
| V2CH | VLDL-2 Subclass | Cholesterol | <0.0001 | 0.0040 |
| V3CH | VLDL-3 Subclass | Cholesterol | <0.0001 | 0.0336 |
| V4CH | VLDL-4 Subclass | Cholesterol | 0.0029 | 0.0006 |
| V5CH | VLDL-5 Subclass | Cholesterol | 0.0400 | 0.0002 |
| VLCH | VLDL Class | Cholesterol | <0.0001 | 0.0052 |
| H4FC | HDL-4 Subclass | Free Cholesterol | <0.0001 | 0.0071 |
| L1FC | LDL-1 Subclass | Free Cholesterol | <0.0001 | 0.0003 |
| L3FC | LDL-3 Subclass | Free Cholesterol | <0.0001 | 0.0386 |
| L4FC | LDL-4 Subclass | Free Cholesterol | 0.0003 | 0.0071 |
| LDFC | LDL Class | Free Cholesterol | 0.0003 | 0.0081 |
| V2FC | VLDL-2 Subclass | Free Cholesterol | <0.0001 | 0.0243 |
| V4FC | VLDL-4 Subclass | Free Cholesterol | 0.0039 | <0.0001 |
| L1PN | LDL-1 | Particle Number | <0.0001 | <0.0001 |
| L2PN | LDL-2 | Particle Number | <0.0001 | 0.0016 |
| L3PN | LDL-3 | Particle Number | <0.0001 | <0.0001 |
| VLPN | VLDL | Particle Number | <0.0001 | 0.0292 |
| H4PL | HDL-4 Subclass | Phospholipids | 0.0091 | 0.0023 |
| IDPL | IDL Class | Phospholipids | 0.0002 | 0.0002 |
| L1PL | LDL-1 Subclass | Phospholipids | <0.0001 | <0.0001 |
| L2PL | LDL-2 Subclass | Phospholipids | <0.0001 | 0.0052 |
| L3PL | LDL-3 Subclass | Phospholipids | <0.0001 | <0.0001 |
| LDPL | LDL Class | Phospholipids | 0.0028 | 0.0026 |
| V2PL | VLDL-2 Subclass | Phospholipids | <0.0001 | 0.0131 |
| V3PL | VLDL-3 Subclass | Phospholipids | <0.0001 | 0.0039 |
| V4PL | VLDL-4 Subclass | Phospholipids | <0.0001 | 0.0002 |
| V5PL | VLDL-5 Subclass | Phospholipids | <0.0001 | 0.0018 |
| VLPL | VLDL Class | Phospholipids | <0.0001 | 0.0454 |
| H1TG | HDL-1 Subclass | Triglycerides | 0.0173 | <0.0001 |
| H2TG | HDL-2 Subclass | Triglycerides | 0.0003 | <0.0001 |
| H3TG | HDL-3 Subclass | Triglycerides | <0.0001 | 0.0028 |
| HDTG | HDL Class | Triglycerides | 0.0012 | <0.0001 |
| L3TG | LDL-3 Subclass | Triglycerides | 0.0402 | 0.0010 |
| V4TG | VLDL-4 Subclass | Triglycerides | <0.0001 | 0.0243 |
| V5TG | VLDL-5 Subclass | Triglycerides | 0.0002 | 0.0116 |

## Supplementary table 5 – Results from 2-way ANOVA of each metabolite between 30 second ischemia and control groups. Selected here are all metabolites that had a significant time and group difference. LDL – Low Density Lipoprotein, HDL – High Density Lipoprotein, VLDL – Very Low Density Lipoprotein, IDL - Intermediate Density Lipoprotein

| **Metabolite** | **Subclass** | **Compound** | **Difference over time** | **Difference between groups** |
| --- | --- | --- | --- | --- |
| **Non-lipids** |  |  |  |  |
| Trimethylamine-N-oxide | Amino acid |  | <0.0001 | 0.0366 |
| Glycine | Amino acid |  | <0.0001 | <0.0001 |
| Histidine | Amino acid |  | <0.0001 | 0.0050 |
| N,N-Dimethylglycine | Amino acid |  | 0.0062 | 0.0006 |
| Succinic acid | Organic acid |  | 0.0003 | 0.0099 |
| Pyruvic acid | Organic acid |  | <0.0001 | 0.0001 |
| **Lipoproteins** |  |  |  |  |
| H1A1 | HDL-1 Subclass | Apolipoprotein-A1 | 0.0075 | <0.0001 |
| H4A1 | HDL-4 Subclass | Apolipoprotein-A1 | 0.0002 | 0.0180 |
| H1A2 | HDL-1 Subclass | Apolipoprotein-A2 | 0.0006 | <0.0001 |
| H4A2 | HDL-4 Subclass | Apolipoprotein-A2 | 0.0023 | 0.0023 |
| L3AB | LDL-3 Subclass | Apolipoprotein-B100 | 0.0007 | 0.0109 |
| VLAB | VLDL Class | Apolipoprotein-B100 | <0.0001 | 0.0011 |
| L3CH | LDL-3 Subclass | Cholesterol | 0.0008 | 0.0093 |
| V1CH | VLDL-1 Subclass | Cholesterol | <0.0001 | <0.0001 |
| V2CH | VLDL-2 Subclass | Cholesterol | <0.0001 | 0.0007 |
| V3CH | VLDL-3 Subclass | Cholesterol | 0.0364 | <0.0001 |
| VLCH | VLDL Class | Cholesterol | <0.0001 | <0.0001 |
| H2FC | HDL-2 Subclass | Free Cholesterol | 0.0221 | 0.0090 |
| L3FC | LDL-3 Subclass | Free Cholesterol | <0.0001 | 0.0007 |
| V1FC | VLDL-1 Subclass | Free Cholesterol | <0.0001 | 0.0008 |
| V2FC | VLDL-2 Subclass | Free Cholesterol | <0.0001 | 0.0011 |
| V3FC | VLDL-3 Subclass | Free Cholesterol | <0.0001 | <0.0001 |
| VLFC | VLDL Class | Free Cholesterol | <0.0001 | 0.0036 |
| L3PN | LDL-3 | Particle Number | 0.0007 | 0.0109 |
| VLPN | VLDL | Particle Number | <0.0001 | 0.0011 |
| L3PL | LDL-3 Subclass | Phospholipids | 0.0001 | 0.0111 |
| V1PL | VLDL-1 Subclass | Phospholipids | <0.0001 | <0.0001 |
| V2PL | VLDL-2 Subclass | Phospholipids | <0.0001 | 0.0021 |
| V3PL | VLDL-3 Subclass | Phospholipids | <0.0001 | 0.0008 |
| V4PL | VLDL-4 Subclass | Phospholipids | 0.0371 | 0.0014 |
| VLPL | VLDL Class | Phospholipids | <0.0001 | 0.0049 |
| H1TG | HDL-1 Subclass | Triglycerides | <0.0001 | <0.0001 |
| H3TG | HDL-3 Subclass | Triglycerides | <0.0001 | 0.0040 |
| H4TG | HDL-4 Subclass | Triglycerides | 0.0001 | <0.0001 |
| IDTG | IDL Class | Triglycerides | <0.0001 | 0.0028 |
| TPTG | Total Plasma | Triglycerides | <0.0001 | 0.0025 |
| V1TG | VLDL-1 Subclass | Triglycerides | <0.0001 | 0.0105 |
| V3TG | VLDL-3 Subclass | Triglycerides | <0.0001 | 0.0151 |
| V4TG | VLDL-4 Subclass | Triglycerides | <0.0001 | 0.0107 |
| VLTG | VLDL Class | Triglycerides | <0.0001 | 0.0044 |

## Supplementary Table 6 – Results from 2-way ANOVA of each metabolite between 60 second ischemia and control groups. Selected here are all metabolites that had a significant time and group difference. LDL – Low Density Lipoprotein, HDL – High Density Lipoprotein, VLDL – Very Low Density Lipoprotein, IDL - Intermediate Density Lipoprotein

| **Name of metabolite** | **Subclass** | **Compound** | **Difference over time** | **Difference between groups** |
| --- | --- | --- | --- | --- |
| **Non-lipids** |  |  |  |  |
| Trimethylamine-N-oxide | Amino acid |  | <0.0001 | 0.0036 |
| Glycine | Amino acid |  | <0.0001 | 0.0004 |
| Leucine | Amino acid |  | 0.0074 | 0.0491 |
| N,N-Dimethylglycine | Amino acid |  | 0.0467 | 0.0009 |
| 3-Hydroxybutyric acid | Organic acid |  | <0.0001 | 0.0118 |
| Pyruvic acid | Organic acid |  | 0.0224 | 0.0007 |
| **Lipoproteins** |  |  |  |  |
| H4A1 | HDL-4 Subclass | Apolipoprotein-A1 | <0.0001 | 0.0037 |
| H1A2 | HDL-1 Subclass | Apolipoprotein-A2 | 0.0044 | 0.0151 |
| H4A2 | HDL-4 Subclass | Apolipoprotein-A2 | 0.0003 | 0.0118 |
| L2AB | LDL-2 Subclass | Apolipoprotein-B100 | 0.0007 | <0.0001 |
| L3AB | LDL-3 Subclass | Apolipoprotein-B100 | 0.0007 | <0.0001 |
| L5AB | LDL-5 Subclass | Apolipoprotein-B100 | 0.0025 | 0.0005 |
| VLAB | VLDL Class | Apolipoprotein-B100 | <0.0001 | <0.0001 |
| L1CH | LDL-1 Subclass | Cholesterol | <0.0001 | 0.0151 |
| L2CH | LDL-2 Subclass | Cholesterol | 0.0010 | <0.0001 |
| L3CH | LDL-3 Subclass | Cholesterol | 0.0009 | <0.0001 |
| L5CH | LDL-5 Subclass | Cholesterol | 0.0036 | 0.0001 |
| V1CH | VLDL-1 Subclass | Cholesterol | <0.0001 | <0.0001 |
| V2CH | VLDL-2 Subclass | Cholesterol | <0.0001 | 0.0001 |
| V3CH | VLDL-3 Subclass | Cholesterol | 0.0388 | <0.0001 |
| VLCH | VLDL Class | Cholesterol | <0.0001 | <0.0001 |
| L2FC | LDL-2 Subclass | Free Cholesterol | <0.0001 | 0.0188 |
| L3FC | LDL-3 Subclass | Free Cholesterol | <0.0001 | <0.0001 |
| L4FC | LDL-4 Subclass | Free Cholesterol | 0.0090 | <0.0001 |
| LDFC | LDL Class | Free Cholesterol | 0.0061 | <0.0001 |
| V1FC | VLDL-1 Subclass | Free Cholesterol | <0.0001 | <0.0001 |
| V2FC | VLDL-2 Subclass | Free Cholesterol | <0.0001 | <0.0001 |
| V3FC | VLDL-3 Subclass | Free Cholesterol | <0.0001 | <0.0001 |
| V5FC | VLDL-5 Subclass | Free Cholesterol | 0.0013 | <0.0001 |
| VLFC | VLDL Class | Free Cholesterol | <0.0001 | <0.0001 |
| L2PN | LDL-2 | Particle Number | 0.0007 | <0.0001 |
| L3PN | LDL-3 | Particle Number | 0.0007 | <0.0001 |
| L5PN | LDL-5 | Particle Number | 0.0025 | 0.0005 |
| VLPN | VLDL | Particle Number | <0.0001 | <0.0001 |
| H4PL | HDL-4 Subclass | Phospholipids | 0.0029 | 0.0071 |
| L2PL | LDL-2 Subclass | Phospholipids | 0.0001 | <0.0001 |
| L3PL | LDL-3 Subclass | Phospholipids | 0.0002 | <0.0001 |
| L5PL | LDL-5 Subclass | Phospholipids | 0.0007 | <0.0001 |
| LDPL | LDL Class | Phospholipids | 0.0314 | <0.0001 |
| V1PL | VLDL-1 Subclass | Phospholipids | <0.0001 | <0.0001 |
| V2PL | VLDL-2 Subclass | Phospholipids | <0.0001 | <0.0001 |
| V3PL | VLDL-3 Subclass | Phospholipids | <0.0001 | <0.0001 |
| V4PL | VLDL-4 Subclass | Phospholipids | 0.0314 | <0.0001 |
| V5PL | VLDL-5 Subclass | Phospholipids | <0.0001 | 0.0113 |
| VLPL | VLDL Class | Phospholipids | <0.0001 | <0.0001 |
| H2TG | HDL-2 Subclass | Triglycerides | 0.0025 | 0.0075 |
| H3TG | HDL-3 Subclass | Triglycerides | <0.0001 | <0.0001 |
| H4TG | HDL-4 Subclass | Triglycerides | <0.0001 | <0.0001 |
| HDTG | HDL Class | Triglycerides | 0.0018 | 0.0161 |
| IDTG | IDL Class | Triglycerides | <0.0001 | <0.0001 |
| L4TG | LDL-4 Subclass | Triglycerides | 0.0159 | <0.0001 |
| L5TG | LDL-5 Subclass | Triglycerides | <0.0001 | 0.0001 |
| TPTG | Total Plasma | Triglycerides | <0.0001 | <0.0001 |
| V1TG | VLDL-1 Subclass | Triglycerides | <0.0001 | <0.0001 |
| V2TG | VLDL-2 Subclass | Triglycerides | <0.0001 | <0.0001 |
| V3TG | VLDL-3 Subclass | Triglycerides | <0.0001 | <0.0001 |
| V4TG | VLDL-4 Subclass | Triglycerides | 0.0001 | <0.0001 |
| V5TG | VLDL-5 Subclass | Triglycerides | 0.0107 | 0.0006 |
| VLTG | VLDL Class | Triglycerides | <0.0001 | <0.0001 |

## Supplementary Fig.1 – Difference between the 60 second ischemia group, 30 second ischemia group and control for the main lipoprotein components. Red – 60 seconds ischemia, Blue – 30 seconds ischemia, yellow – control


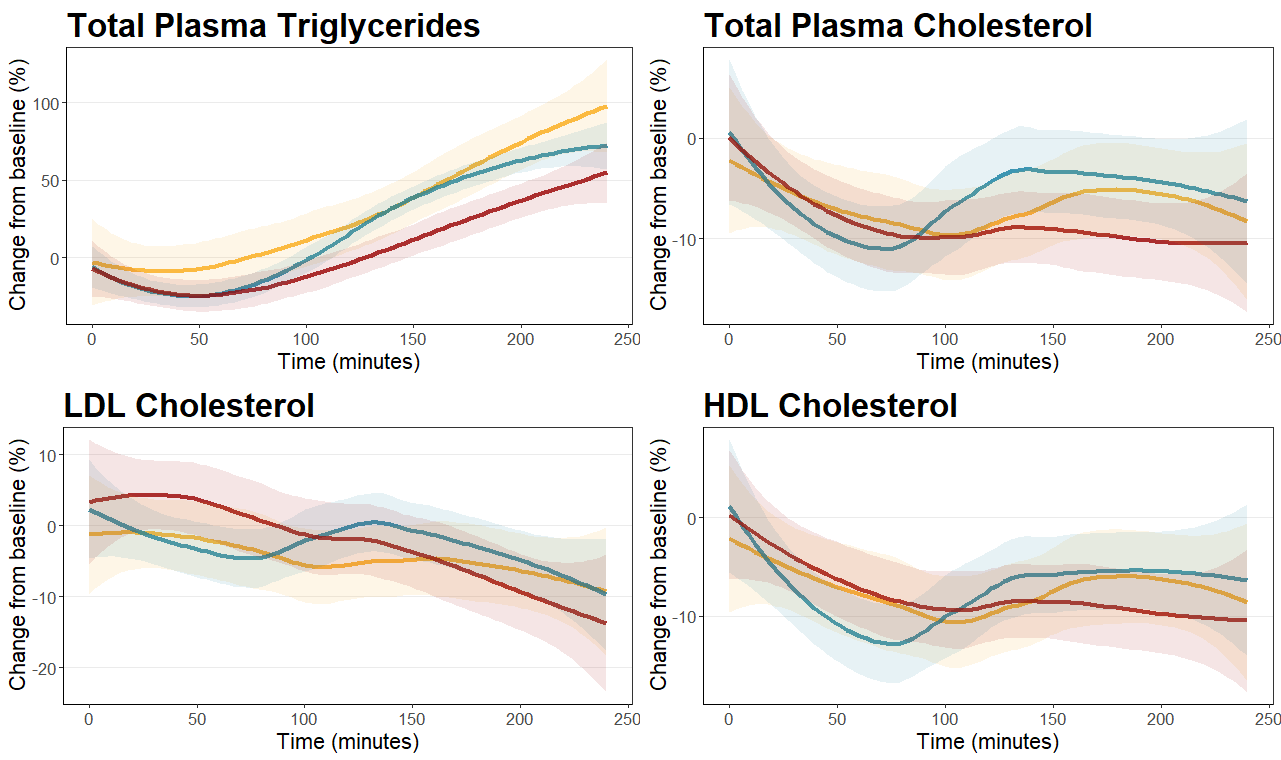


## Supplementary Fig.2 – Difference between the 60 second ischemia group, 30 second ischemia group and control for selected metabolites of the Tricarboxylic Acid Cycle. Red – 60 seconds ischemia, blue – 30 seconds ischemia, yellow – control


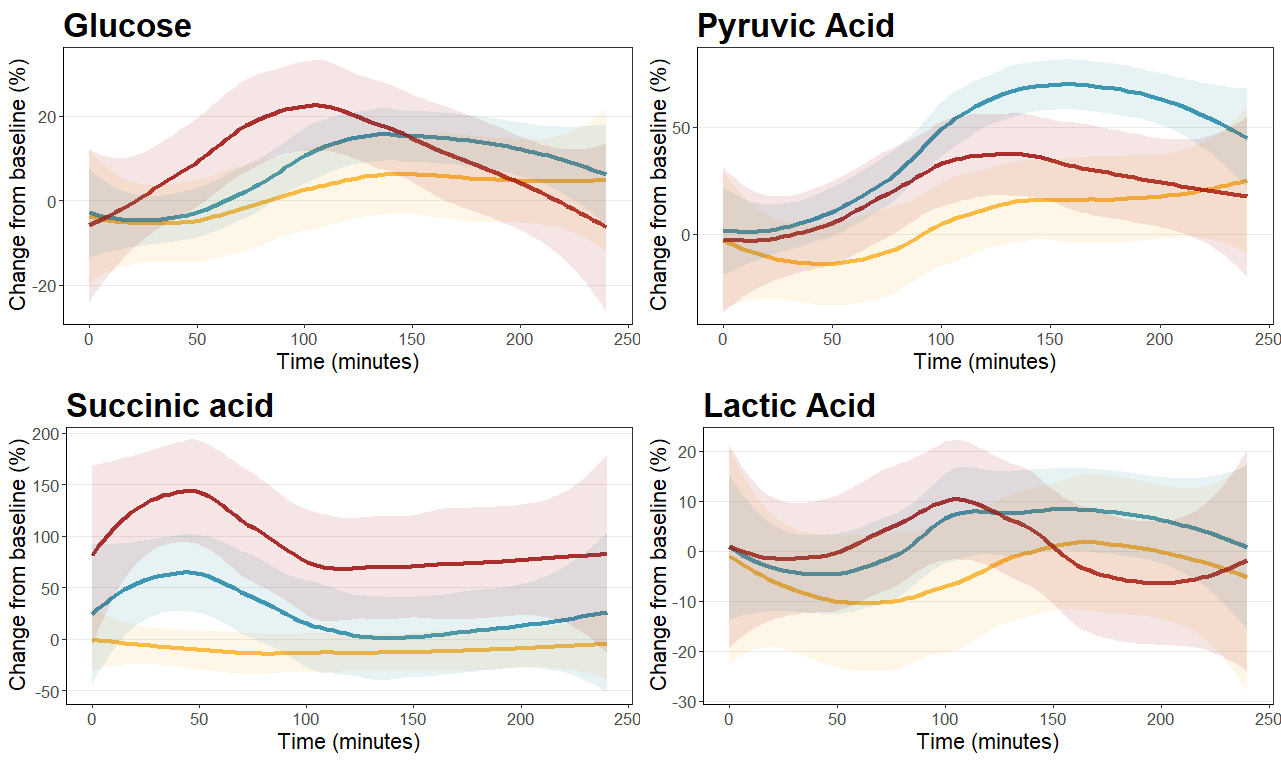


## Supplementary Fig.3 – Quantification of selected metabolites from the principal component analyses for time 0 to 15 minutes (A) and 0 to 60 minutes (B) for all groups. Yellow – control, blue – 30 seconds ischemia, red – 60 seconds ischemia, green – 90 seconds ischemia. LDL – Low Density Lipoprotein, HDL – High Density Lipoprotein.


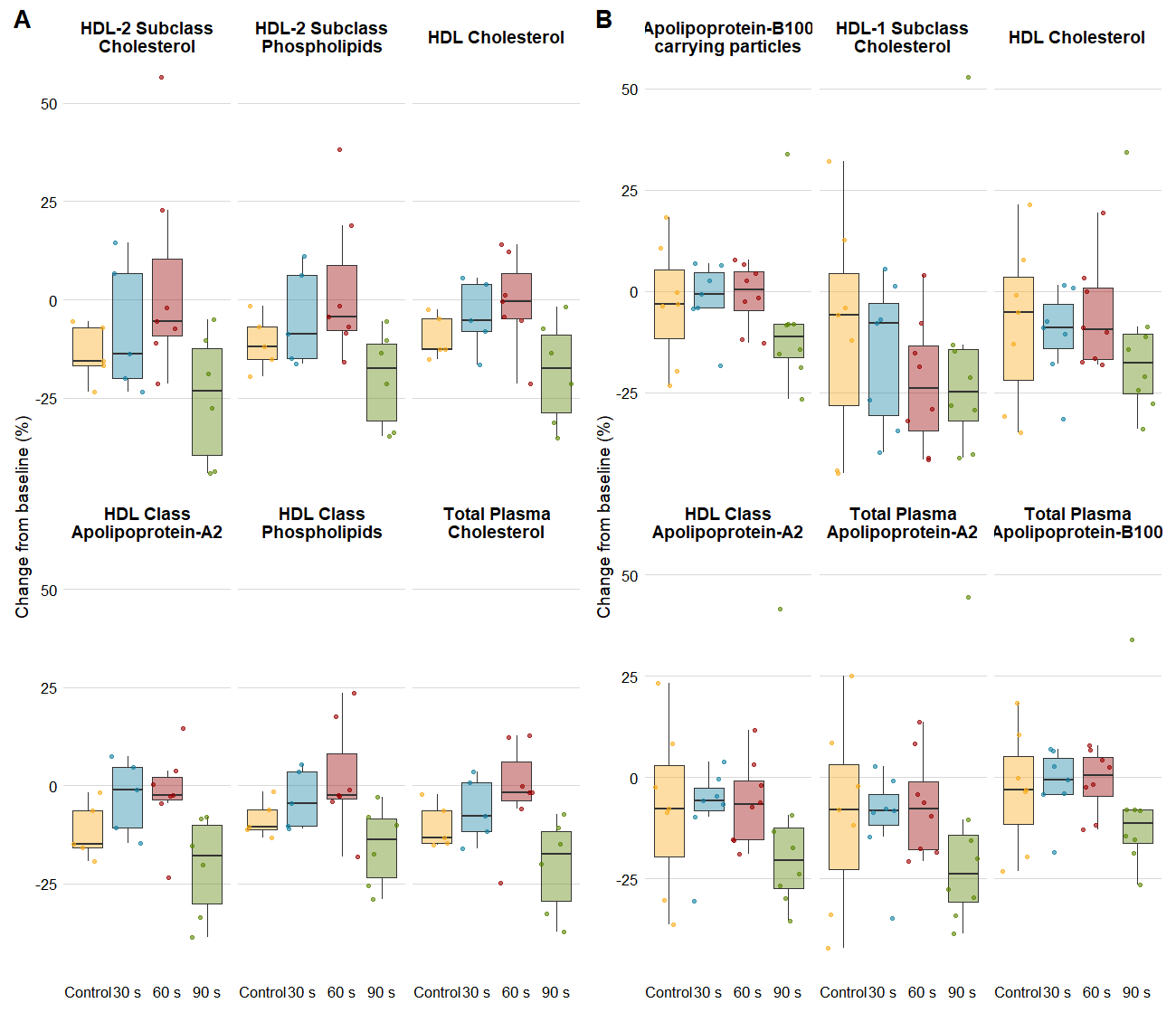

Supplement: Supplementary file 1 — Supplementary Material 1 [file 11306_2023_1999_MOESM1_ESM.docx]
